# Movements of transposable elements contribute to the genomic plasticity and species diversification in an asexually reproducing nematode pest

**DOI:** 10.1111/eva.13246

**Published:** 2021-05-15

**Authors:** Djampa K. L. Kozlowski, Rahim Hassanaly‐Goulamhoussen, Martine Da Rocha, Georgios D. Koutsovoulos, Marc Bailly‐Bechet, Etienne G. J. Danchin

**Affiliations:** ^1^ Université Côte d’Azur INRAE CNRS ISA Sophia Antipolis France

**Keywords:** bioinformatics/phyloinformatics, contemporary evolution, evolution of sex, genomics/proteomics, host–parasite interactions, hybridization, transposons

## Abstract

Despite reproducing without sexual recombination, *Meloidogyne incognita* is an adaptive and versatile phytoparasitic nematode. This species displays a global distribution, can parasitize a large range of plants, and can overcome plant resistance in a few generations. The mechanisms underlying this adaptability remain poorly known. At the whole‐genome level, only a few single nucleotide variations have been observed across different geographical isolates with distinct ranges of compatible hosts. Exploring other factors possibly involved in genomic plasticity is thus important. Transposable elements (TEs), by their repetitive nature and mobility, can passively and actively impact the genome dynamics. This is particularly expected in polyploid hybrid genomes such as the one of *M*. *incognita*. Here, we have annotated the TE content of *M*. *incognita*, analyzed the statistical properties of this TE landscape, and used whole‐genome pool‐seq data to estimate the mobility of these TEs across twelve geographical isolates, presenting variations in ranges of compatible host plants. DNA transposons are more abundant than retrotransposons, and the high similarity of TE copies to their consensus sequences suggests they have been at least recently active. We have identified loci in the genome where the frequencies of presence of a TE showed substantial variations across the different isolates. Overall, variations in TE frequencies across isolates followed their phylogenetic divergence, suggesting TEs participate in the species diversification. Compared with the *M*. *incognita* reference genome, we detected isolate and lineage‐specific de novo insertion of some TEs, including within genic regions or in the upstream regulatory regions. We validated by PCR the insertion of some of these TEs inside genic regions, confirming TE movements have possible functional impacts. Overall, we show DNA transposons can drive genomic plasticity in *M*. *incognita* and their role in genome evolution of other parthenogenetic animal deserves further investigation.

## 1 INTRODUCTION

Agricultural pests cause substantial yield loss to the worldwide life‐sustaining production (Savary et al., 2019) and threaten the survival of different communities in developing countries. With a constantly growing human population, it becomes more and more crucial to reduce the loss caused by these pests while limiting the impact on the environment. In this context, understanding how pests evolve and adapt both to the control methods deployed against them and to a changing environment is essential. Among Metazoa, nematodes and insects are the most destructive agricultural pests. Nematodes alone are responsible for crop yield losses of ca. 11%, representing up to 100 billion € economic loss annually (Agrios, 2005; McCarter, 2009). The most problematic nematodes to worldwide agriculture belong to the genus *Meloidogyne* (Jones et al., 2013) and are commonly named root‐knot nematodes (RKN) owing to the gall symptoms their infection leaves on the roots. The RKN species showing the wider geographical distribution and infecting the broadest diversity of plants reproduce asexually via mitotic parthenogenesis (Castagnone‐Sereno & Danchin, 2014; Trudgill & Blok, 2001). This observation seems counterintuitive as animal species with strictly asexual reproduction are deemed less adaptive than their sexual relatives, are quite rare, and occupy shallow branches in the animal tree of life (Rice, 2002). In the absence of sexual reproduction, the combination of beneficial alleles from different individuals is impossible and the efficiency of selection is reduced due to linkage between conflicting alleles (Glémin et al., 2019; Hill & Robertson, 1966; Kondrashov, 1988; Muller, 1964). Consistent with the theory, population genomic analyses have revealed the efficacy of purifying selection is reduced in *M*. *incognita* as compared to two outcrossing species in the *Caenorhabditis* genus (Koutsovoulos, Marques et al., 2020).

Previous comparative genomic studies have shown the genomes of the most devastating RKN are polyploid because of hybridization events (Blanc‐Mathieu et al., 2017; Szitenberg et al., 2017). In the parthenogenetic RKN *M*. *incognita*, the gene copies resulting from allopolyploidy diverge not only at the nucleotide level but also in their expression patterns, suggesting this peculiar genome structure could support a diversity of functions and might be involved in their higher parasitic success despite the absence of sexual reproduction (Blanc‐Mathieu et al., 2017). This hypothesis seems consistent with the “general‐purpose genotype” concept, which proposes successful parthenogens have a generalist genotype with good fitness in a variety of environments (Vrijenhoek & Parker, 2009). An alternative non‐mutually exclusive hypothesis is the “frozen niche variation” concept, which proposes parthenogens are more successful in stable environments because they have a frozen genotype adapted to this specific environment (Vrijenhoek & Parker, 2009). Interestingly, the frequency of parthenogenetic invertebrates is higher in agricultural pests, probably because the anthropized environments in which they live are more stable and uniform (Hoffmann et al., 2008).

However, although a general‐purpose genotype brought by hybridization might contribute to the wide host range and geographical distribution of these RKNs, this alone cannot explain how these parthenogenetic species evolve and adapt to new hosts or environments. For instance, initially avirulent populations of some of these RKN, controlled by a resistance gene in a tomato, are able to overcome the plant resistance in a few generations, leading to virulent subpopulations, in controlled laboratory experiments (Castagnone‐Sereno, 2006; Castagnone‐Sereno et al., 1994). Emergence of virulent populations not controlled anymore by resistance genes has also been reported in the field (Barbary et al., 2015).

The mechanisms underlying the adaptability of parthenogenetic RKN remain elusive. Previous population genomic analyses identified only a few single nucleotide variations (SNV) by comparing different Brazilian and other *M*. *incognita* isolates across the world showing distinct ranges of host compatibility (Koutsovoulos, Marques et al., 2020). Furthermore, the few identified SNV showed no significant correlation with either the geographical location, the host range, or the currently infected crop species. However, these SNV could be used as markers to confirm the absence of sexual meiotic recombination in *M*. *incognita*. Thus, the low nucleotide variability that was observed between isolates is probably not the main driver of the genomic plasticity underlying the adaptability and diversification of *M*. *incognita*.

Consistent with these views, convergent gene copy‐number variations were observed following resistance breaking down by two originally avirulent populations of *M*. *incognita* from distinct geographical origins (Castagnone‐Sereno et al., 2019). The mechanisms supporting these gene copy numbers and other genomic variations possibly involved in the adaptive evolution of *M*. *incognita* remain to be described.

Transposable elements (TEs), by their repetitive and mobile nature, can both passively and actively impact genome plasticity. Being repetitive, they can be involved in genomic rearrangements leading to loss of genomic portions or expansion of gene copy numbers. Being mobile, they can insert in coding or regulatory regions and have a functional impact on the gene expression or gene structure/function itself. For instance, TE insertions have been shown to affect gene expression in a species‐specific manner in amniotes (Zeng et al., 2018) and, in rodents, TE insertions account for ca. 20% of gene expression profile divergence between mice and rats (Pereira et al., 2009). At shorter evolutionary scales, differential presence/absence of TE across *Arabidopsis* populations revealed rare variants associated with extremes of gene expression (Stuart et al., 2016). TE insertions in coding regions can disrupt a gene, and this disruption might eventually have an adaptive effect. For example, a TE insertion has caused disruption of a Phytochrome A gene in some soybean strains, which caused photoperiod insensitivity and was in turn associated with adaptation to high latitudes in Japan (Kanazawa et al., 2009). Moreover, in *Drosophila*, insertion of a TE in the *CHKov1* gene caused four new alternative transcripts and this modification is associated with resistance to insecticide and viral infection (Aminetzach et al., 2005; Magwire et al., 2011). In parallel, although TE movements can provide beneficial genomic novelty or plasticity, their uncontrolled activity can also be highly detrimental and put the organism at risk. For instance, some human diseases such as hemophilia (Kazazian et al., 1988) or cancers (Miki et al., 1992) are caused by TE insertions in coding or regulatory regions.

Concerning agricultural pests themselves, TEs are a major player of adaptive genome evolution by both passively and actively impacting the genome structure and sequence in some fungal phytopathogens (Faino et al., 2016). Whether TEs also play an important role in the genome plasticity and possibly adaptive evolution of parasitic animals, engaged in a continuous arms race with their hosts, remains poorly known. According to the Red Queen hypothesis, host–parasite arms race is a major justification for the prevalence of otherwise costly sexual reproduction (Lively, 2010) and, in the absence of sex, other mechanisms should provide the necessary plasticity to sustain this arms race.

From an evolutionary point of view, the parthenogenetic root‐knot nematode *M*. *incognita* represents an interesting model to study the activity of TEs and their impact on the genome, including in coding or regulatory regions. Indeed, being a plant parasite, *M*. *incognita* is engaged in an arms race with the plant defense systems and point mutations alone are not expected to be a major mechanism supporting adaptation in this species (Koutsovoulos, Marques et al., 2020).

In a broader perspective, little is known yet about the TE dynamics in nematode genomes and their possible impact on adaptive evolution, including in the model *Caenorhabditis elegans*, despite being the first sequenced animal genome (The C. elegans Genome Sequencing Consortium, 1998). Transposition activity of Tc1 TIR element was shown to be positively linked to the overall mutation rate in *C*. *elegans* mutator strains, one of which is characterized by high transposition in the germline, hence constituting a considerable evolutionary force (Bégin & Schoen, 2007). However, these results may be hindered by the fact that, in wild‐type *C*. *elegans*, although Tc1 excision frequency is substantial in somatic cells, it is negligible in the germ cells (Emmons & Yesner, 1984).

Besides Tc1, a more comprehensive analysis using population genomic approach in *C*. *elegans* represents the most advanced study of the TE dynamics in this species to date (Laricchia et al., 2017). By analyzing hundreds of wild populations of *C*. *elegans*, the authors have shown a substantial level of activity for multiple TE families in these genomes compared with the N2 reference strain. The study points at a population‐wide variability of this activity, and, surprisingly, toward little evident phenotypic effect of this activity, even when TEs were found inserted into coding sequences. Concerning the possible functional impact of TE activity in nematodes, an investigation of TE expression in *C*. *elegans* germline in a single‐cell framework has shown significant differences between the expression pattern of LTR, non‐LTR retroelements, and DNA transposons, associated with differentiated vs. undifferentiated cell types (Ansaloni et al., 2019). These complex cell‐type‐specific differential expression patterns suggest TE activity plays an important role in the *C*. *elegans* embryonic development, although the exact role remains elusive. Overall, while it is now clearly established that TEs are active in *C*. *elegans* and probably contribute to the genome plasticity, their possible functional implication or role in nematode adaptive evolution has not been shown so far.

In this study, we have tested whether movements of TEs could represent a mechanism supporting genome plasticity in *M*. *incognita*, a prerequisite for adaptive evolution. We have reannotated the 183.5‐Mb triploid genome of *M*. *incognita* (Blanc‐Mathieu et al., 2017) for TEs, and using stringent filters, we have only retained those harboring the characteristic features of known retro and DNA transposon orders, hence more likely to be active. We analyzed the statistical properties of the TE content, and the distribution of TE sequence identity levels to their consensuses was used as a reporter of the recentness of their activity. We have then tested whether the frequencies of presence/absence of these TEs across the genome varied between different isolates. To test for variations in frequencies, we have used population genomics data from eleven *M*. *incognita* isolates collected on different crops and locations and showing distinct ranges of compatible hosts (Koutsovoulos, Marques et al., 2020). From the set of TE loci that presented the most contrasted patterns of presence/absence across the isolates, we investigated whether some could represent isolate or lineage‐specific insertions. To estimate the possible functional impact of TE insertions, we checked whether some were inserted within coding or possible regulatory regions. Finally, we validated by PCR assays several of these insertions in coding or regulatory regions, predicted by population genomics data. Overall, our study represents the first estimation of TE activity as a mechanism possibly involved in the genome plasticity and the associated functional impact in the most devastating nematode to worldwide agriculture. Besides *C*. *elegans*, little was known about the role of TE in the genome dynamics of *Nematoda*, one of the most species‐rich animal phylum. Because this study focuses on an allopolyploid and parthenogenetic animal species, it also opens new evolutionary perspectives on the fate and potential adaptive impact of TEs in these singular organisms.

## 2 MATERIALS AND METHODS

### 2.1 Material

#### 2.1.1 The genome of *Meloidogyne incognita*

We used the genome assembly published in (Blanc‐Mathieu et al., 2017) as a reference for TE prediction and annotation (ENA assembly accession GCA_900182535, bioproject PRJEB8714) and for read‐mapping of the different geographical isolates (Koutsovoulos, Marques et al., 2020), used for prediction of TE presence frequencies.

Briefly, the triploid *M*. *incognita* genome is 183.5 Mb long with ~12,000 scaffolds and a N50 length of ~38 kb. Although the genome is triploid, because of the high nucleotide divergence between the genome copies (8% on average), most of these genome copies have been correctly separated during genome assembly, which can be considered effectively haploid (Blanc‐Mathieu et al., 2017; Koutsovoulos, Marques et al., 2020). This reference genome originally came from a *M*. *incognita* population from the Morelos region of Mexico and was reared on tomato plants from the offspring of one single female in our laboratory.

#### 2.1.2 The genome of *Caenorhabditis elegans*

We used the *C*. *elegans* genome (The C. elegans Genome Sequencing Consortium, 1998) assembly (PRJNA13758) to perform its repeatome prediction and annotation and compare our results with the literature as a methodological validation.

#### 2.1.3 Genome reads for 12 *Meloidogyne incognita* geographical isolates

To predict the presence frequencies at TE loci across different *M*. *incognita* isolates, we used whole‐genome sequencing data from pools of individuals from 12 different geographical regions (Figure S1; Table S1). One pool corresponds to the Morelos isolates used to produce the *M*. *incognita* reference genome itself, as described above. The 11 other pools correspond to different geographical isolates across Brazil as described in Koutsovoulos, Marques et al. (2020).

All the samples were reared from the offspring of one single female and multiplied on tomato plants. Then, approximately 1 million individuals were pooled and sequenced by Illumina paired‐end reads (2 × 150 bp). Library sizes vary between 74 and 76 million reads (Koutsovoulos, Marques et al., 2020).

We used cutadapt‐1.15 (Martin, 2011) to trim adapters, discard small reads, and trim low‐quality bases in read boundaries (–max‐n = 5 ‐q 20,20 ‐m 51 ‐j 32 ‐a AGATCGGAAGAGCACACGTCTGAACTCCAGTCA ‐A AGATCGGAAGAGCGTCGTGTAGGGAAAGAGTGT). Then, for each library, we performed a fastqc v‐0.11.8 (Andrews, 2010) analysis to evaluate the quality of the reads. FastQC result analyses showed that no additional filtering or cleaning step was needed and no further read was discarded.

### 2.2 Methods

We performed the statistical analyses and the graphical representations using R’ v‐3.6.3 and the following libraries: ggplot2, cowplot, reshape2, ggpubr, phangorn, tidyverse, and ComplexUpset. All codes and analysis workflows are publicly available in the INRAE Dataverse (Kozlowski, 2020a, 2020d; Kozlowski et al., 2020). For experimental validations, see Kozlowski et al. (2020). A diagram recapitulating the main steps of the analysis has been provided in supplementary Figure S2, as well as a decision tree summarizing the polymorphism characterization (Figure S3).

#### 2.2.1 *Meloidogyne incognita* and *Caenorhabditis elegans* repeatome predictions and annotations

We predicted and annotated the *M*. *incognita* and *C*. *elegans* repeatomes following the same protocol as thoroughly explained in Koutsovoulos, Poullet et al. (2020). We define the repeatome as all the repeated sequences in the genome, excluding simple sequence repeats (SSR) and microsatellites. Then, following the above‐mentioned protocol, we further analyzed each repeatome to retain only annotations with canonical signatures of transposable elements (TEs).

Below, we briefly explain each step and describe protocol adjustments.

#### 2.2.2 Genome preprocessing

Unknown nucleotides “Ns” encompass 1.81% of *the M*. *incognita* reference genome and need to be trimmed before repeatome predictions. We created a modified version of the genome by splitting it at *N* stretches of length 11 or more and then trimming all *N*, using dbchunk.py from the REPET package (Flutre et al., 2011; Quesneville et al., 2005). As this increases genome fragmentation and may, in turn, lead to false positives in TE detection, we only kept chunks of length above the L90 chunk length threshold, which is 4891 bp. This modified version of the genome was only used to perform the de novo prediction of the TE consensus library (below). The TE annotation was performed on the original whole reference genome.

The *C*. *elegans* reference genome was entirely resolved (no *N*), at the chromosome scale. Hence, we used the whole assembly as is to perform the de novo prediction analysis.

#### 2.2.3 De novo prediction: constituting draft TE consensus libraries

For each species, we used the TEdenovo pipeline from the REPET package to generate a draft TE consensus library.

Briefly, TEdenovo pipeline (i) realizes a self‐alignment of the input genome to detect repetitions, (ii) clusters the repetitions, (iii) performs multiple alignments from the clustered repetitions to create consensus sequences, and (iv) eventually classifies the consensus sequence following Wicker's classification (Wicker et al., 2007) using structural and homology‐based information. One of the most critical steps of this process concerns the clustering of the repetitions as it requires prior knowledge about assembly ploidy and phasing quality.

We ran the analysis considering the modified *M*. *incognita* reference assembly previously described as triploid and set the “minNbSeqPerGroup” parameter to 7 (i.e., 2n+1). As the *C*. *elegans* assembly was haploid, we set the same parameter to 3.

All the remaining parameter values set in these analyses can be found in the TEdenovo configuration files (Kozlowski, 2020a).

#### 2.2.4 Automated curation of the TE consensus libraries

To limit the redundancy in the previously created TE consensus libraries and the false positives, we performed an automated curation step. Briefly, for each species, (i) we performed a minimal annotation (steps 1, 2, 3, 7 of TEannot) of their genome with their respective draft TE consensus libraries, and (ii) only retained consensus sequences with at least one full‐length copy (FLC) annotated in the genome. All parameter values are described in the configuration files available in Kozlowski (2020a).

#### 2.2.5 Repeatome annotation

For each species, we performed a full annotation (steps 1, 2, 3, 4, 5, 7, and 8) of their genome with their respective cleaned TE consensus libraries using TEannot from the REPET package. The obtained repeatome annotations (excluding SSR and microsatellites) were exported for further analyses. All parameter values are described in the configuration files available in Kozlowski (2020a).

#### 2.2.6 Repeatome postprocessing: identifying annotations with canonical signatures of TEs

Using in‐house scripts (Kozlowski, 2020a), we analyzed REPET outputs to retain annotations with signatures of canonical transposable elements (TEs) from the rest of the repeatomes. The same parameters were set for *M*. *incognita* and *C*. *elegans*. Briefly, for each species, we only conserved TE annotations (i) classified as retrotransposons or DNA transposons, (ii) longer than 250 bp, (iii) sharing more than 85% identity with their consensus sequence (below this value, there is uncertainty on the correspondence between a TE annotation and its consensus if closely related consensuses sequences exist), (iv) covering more than 33% of their consensus sequence length (threshold value allowing the exclusion of a significant number of fragmentary copies), (v) first aligning with their consensus sequence in a BLAST analysis against the TE consensus library, and (vi) not overlapping with other annotations.

TE annotations respecting all the described criterion were referred to as canonical TE annotations.

#### 2.2.7 Putative transposition machinery identification (*Meloidogyne incognita* only)

We analyzed the *M*. *incognita* predicted proteome and transcriptome (Blanc‐Mathieu et al., 2017) and cross‐referenced the obtained information with the canonical TE annotation to identify TE‐containing genes putatively involved in the transposition machinery and evaluate TE‐related gene expression levels in comparison with the rest of the genes in the genome.

#### 2.2.8 Finding genes coding for proteins with TE‐related HMM profiles

We performed an exhaustive HMMprofile search analysis on the whole *M*. *incognita* predicted proteome and then looked for proteins with TE‐related domains. First, we concatenated two HMMprofile libraries into one: Pfram32 (Finn et al., 2016) library and Gypsy DB 2.0 (Llorens et al., 2011), a curated library of HMMprofiles linked to viruses, mobile genetic elements, and genomic repeats. Then, using this concatenated HMM profile library, we performed an exhaustive but stringent HMM profile search on the *M*. *incognita* proteome using hmmscan (‐E 0.00001 ‐‐domE 0.001 ‐‐noali).

Eventually, using in‐house script (Kozlowski, Da Rocha et al., 2020), we selected the best nonoverlapping HMM profiles for each protein and then tagged corresponding genes with TE‐related HMM profiles thanks to a knowledge‐based function from the REPET tool “profileDB4Repet.py.” We kept as genes with TE‐related profiles all the genes with at least one TE‐related HMM profile identified.

#### 2.2.9 Gene expression levels

To determine the *M*. *incognita* protein‐coding gene expression patterns, we used data from a previously published life stage‐specific RNA‐seq analysis of *M*. *incognita* transcriptome during tomato plant infection (Blanc‐Mathieu et al., 2017). This analysis encompassed four different life stages: (i) eggs, (ii) preparasitic second‐stage juveniles (J2), (iii) a mix of late parasitic J2, third‐stage (J3) and fourth‐stage (J4) juveniles, and (iv) adult females, all sequenced in triplicates.

The cleaned RNA‐seq reads were retrieved from the previous analysis and re‐mapped to the *M*. *incognita* annotated genome assembly (Blanc‐Mathieu et al., 2017) using a more recent version of STAR (2.6.1) (Dobin et al., 2013) and the more stringent end‐to‐end option (i.e., no soft clipping) in 2‐passes. Expected read counts were calculated on the predicted genes from the *M*. *incognita* GFF annotation as FPKM values using RSEM (Li & Dewey, 2011) to take into account the multimapped reads via expectation maximization. To reduce amplitude of variations, raw FPKM values were transformed to Log10(FPKM+1), and the median value over the three replicates was kept as a representative value in each life stage. The expression data are available in Danchin and Da Rocha (2020).

Then, for each life stage independently, (i) we ranked the gene expression values and (ii) defined gene expression level corresponding to the gene position in the ranking. We considered as substantially expressed all the genes that presented an expression level ≥ 1st quartile in at least one life stage.

#### 2.2.10 TE annotations with potential transposition machinery

To identify TE annotations including predicted genes involved in transposition machinery (inclusion ≥ 95% of the gene length), we performed the intersection of the canonical TE annotation and the genes annotation BED files (Kozlowski, Da Rocha et al., 2020) using the intersect tool (‐wo ‐s ‐F 0.95) from the bedtools v‐2.27.1 suite (Quinlan & Hall, 2010).

We then cross‐referenced the obtained file with the list of the substantially expressed genes and the list of the TE‐related genes previously produced to identify the TEs containing potential transposition machinery genes and their expression levels.

### 2.3 Evaluation of TE presence frequencies across the different *Meloidogyne incognita* isolates

We used the PoPoolationTE2 v‐1.10.04 pipeline (Kofler et al., 2016) to compute isolate‐related support frequencies of both annotated and de novo TE loci across the 12 *M*. *incognita* geographical isolates previously described. To that end, we performed a “joint” analysis as recommended by the PoPoolationTE2 manual. Briefly, PoPoolationTE2 uses both quantitative and qualitative information extracted from paired‐end (PE) reads mapping on the TE‐annotated reference genome and a set of reference TE sequences to detect signatures of TE polymorphisms and estimate their frequencies in every analyzed isolate. Frequency values correspond to the proportion of reads in an isolate supporting the presence of a copy of the TE at a given locus.

#### 2.3.1 Preparatory work: creating the TE hierarchy and the TE‐merged reference files

We used the canonical TE annotation set created above (Kozlowski, 2020a) and the *M*. *incognita* reference genome to produce the TE‐merged reference file and the TE‐hierarchy file necessary to perform the PoPoolationTE analysis (Kozlowski, 2020d).

We used getfasta and maskfasta commands (default parameters) from the bedtools suite to respectively extract and mask the sequences corresponding to canonical TE annotations in the reference genome. Then, we concatenated both resulting sequences in a “TE‐merged reference” multi fasta file. The “TE‐hierarchy” file was created from the TE annotation file from which it retrieves and stores the TE sequence name, the family, and the TE order for every entry.

#### 2.3.2 Reads mapping

For each *M*. *incognita* isolate library, we mapped forward and reverse reads separately on the "TE‐merged‐references" genome‐TE file using the local alignment algorithm bwa bwasw v‐0.7.17‐r1188 (Li & Durbin, 2009) with the default parameters. The obtained sam alignment files were then converted to bam files using samtools view v‐1.2 (Li et al., 2009).

#### 2.3.3 Restoring paired‐end information and generating the ppileup file

We restored paired‐end information from the previous separate mapping using the sep2pe (‐‐sort) tool from PoPoolationTE2‐v1.10.03. Then, we created the ppileup file using the “ppileup” tool from PoPoolationTE2 with a map quality threshold of 15 (‐‐map‐qual 15).

For every base of the genome, this file summarizes the number of PE read inserts spanning the position (physical coverage) but also the structural status inferred from paired‐end read covering this site.

#### 2.3.4 Estimating target coverage and subsampling the ppileup to a uniform coverage

As noticed by R. Kofler, heterogeneity in physical coverage between populations may lead to discrepancies in TE frequency estimation. Hence, we flattened the physical coverage across the *M*. *incognita* isolates by a subsampling and a rescaling approach.

We first estimated the optimal target coverage to balance information loss and homogeneity using the “stats‐coverage” tool from PoPoolationTE2 (default parameter) and set this value to 15X. We then used the “subsamplePpileup” tool (‐‐target‐coverage 15) to discard positions with a physical coverage below 15X and rescale the coverage of the remaining position to that value.

#### 2.3.5 Identifying signatures of TE polymorphisms

We identified signatures of TE polymorphisms from the previously subsampled file using the “identifySignature” tool following the joint algorithm (‐‐mode joint; ‐‐min‐count 2; ‐‐signature‐window minimumSampleMedian; ‐‐min‐valley minimumSampleMedian).

Then, for each identified site, we estimated TE presence frequencies in each isolate using the “frequency” tool (default parameters). Eventually, we paired up the signatures of TE polymorphisms using “pairupSignatures” tool (‐‐min‐distance −200; ‐‐max‐distance ‐‐ 300 as recommended by R. Kofler), yielding a final list of potential TE polymorphism loci in the reference genome with their associated frequencies for each one of the isolates.

#### 2.3.6 Evaluation of PoPoolationTE2 systematic error rate in the TE frequency estimation

To estimate PoPoolationTE2 systematic error rate in the TE frequency estimation, we ran the same analysis (from the PE information restoration step) but comparing each isolate against itself (12 distinct analyses).

We then analyzed each output individually, measuring the frequency difference between the two “artificial replicates” in all the detected loci with FR signatures (see below for more explanations).

We tested the homogeneity of the frequency difference across the 12 analyses with an ANOVA and concluded that the mean values of the frequency differences between the analysis were not significantly heterogeneous (*p* value = 0.102 > 0.05). Hence, we concatenated the 12 analyses’ frequency difference and set the systematic error rate in the TE frequency estimation to 2 times the standard deviation of the frequency differences, a value of 0.97%.

### 2.4 TE polymorphism analysis

#### 2.4.1 Isolating TE loci with frequency variation across *Meloidogyne incognita* isolates

We parsed PoPoolationTE2 analysis output to identify TE loci with enough evidence to characterize them as polymorphic in frequency across the isolates.

PoPoolationTE2 output informs for each detected locus (i) its position on the reference genome, (ii) its frequency value for every sample of the analysis (e.g., each isolate), and (iii) qualitative information about the reads mapping signatures supporting a TE insertion.

In opposition to separate forward (“F”) or reverse (“R”) signatures, “FR” signatures mean both boundaries of locus are supported by significant physical coverage. Entries with such type of signature are more accurate in terms of frequency and position estimation. Hence, we only retained candidate loci with “FR” signatures. Then, for each locus, we computed the maximal frequency variation between all the isolates and discarded the loci with a frequency difference smaller than the PoPoolationTE2 systematic error rate in the TE‐ frequency estimation we computed (0.97%; see above). We also discarded loci where different TEs were predicted to be inserted. We considered the remaining loci as polymorphic in frequency across the isolates.

#### 2.4.2 Isolates phylogeny

We inferred the *M*. *incognita* isolates phylogeny according to their patterns of polymorphism in TE frequencies.

We first computed a Euclidean distance matrix from the isolate TE frequencies of all the detected polymorphic loci. We then used the distance matrix to construct the phylogenetic tree using the neighbor‐joining (NJ) method (R’ phangorn package v‐2.5.5). We computed nodes’ support values with a bootstrap approach (*n* = 500 replicates) using the boot.phylo function from the ape‐v5.4 R package (Paradis & Schliep, 2019). The boot.phylo function performs a resampling of the frequency matrix (here the matrix with loci in columns, isolates in rows, and values corresponding to the TE presence frequencies).

We also created a phylogenetic tree using the SNV identified within coding regions for all isolates with raxml‐ng v‐0.9.0 (Kozlov et al., 2019) utilizing the model GTR+G+ASC_LEWIS and performing 100 bootstrap replicates. We compared both topologies using Itol v‐4.0 viewer (Letunic & Bork, 2019).

#### 2.4.3 Polymorphism characterization

We exported the polymorphic TE positions as an annotation file, and we used bedtools intersect (‐wao) to perform their intersection with the reference canonical TE annotation. We then cross‐referenced the results with the filtered PoPoolationTE2 output and defined a decision tree to characterize the TE polymorphisms detected by PoPoolationTE2 as “reference TE polymorphism” (ref‐polymorphism), “unannotated,” or “new” loci, as compared to the reference genome (Figure S3).

We considered a reference TE annotation as polymorphic (e.g., ref‐polymorphism locus) if:

- The position of the polymorphism predicted by PoPoolationTE2 falls between the boundaries of the reference TE annotation.
- Both the reference TE annotation and the predicted polymorphism belong to the same TE consensus sequence.
- The TE has a predicted frequency >75% in the reference isolate Morelos.

Canonical TE annotations that did not intersect with polymorphic loci predicted by PoPoolationTE2, or that presented frequency variations <1% across the isolates were considered as nonpolymorphic.

We classified as “new TE loci” all the polymorphic loci for which no canonical TE was predicted by REPET in the reference annotation (polymorphism position is not included in a reference TE annotation), but which were detected with a frequency >25% in at least one isolate different from the reference isolate Morelos, in which the TE frequency should be inferior to 1% and thus considered truly absent in the reference genome.

Finally, we classified as “unannotated TE loci” all the polymorphic loci, which did not correspond to a reference annotation but which were detected with a frequency >25% in the reference isolate Morelos (at least). Polymorphic loci having a frequency between 1% and 25% in Morelos isolate were considered ambiguous and were discarded.

Then, for each TE polymorphism, we investigated the homogeneity of the TE frequency between the isolates. We considered TE frequency was homogeneous between isolates when the maximum frequency variation between isolate was <= to 25%. Above this value, we considered the TE presence frequency was heterogeneous between isolates.

### 2.5 Highly contrasted polymorphic TE loci (HCPTEs): isolation, characterization, and experimental validation

#### 2.5.1 HCPTE isolation

We considered as highly contrasted all the polymorphic loci for which (i) all the isolates had frequency values either <25% or >75%, and (ii) at least one isolate showed a frequency <25%, while another presented a frequency >75%. Polymorphic loci fitting with these requirements were exported as an annotation file in the bed format.

#### 2.5.2 HCPTE possible functional impact

We first identified the genes potentially impacted by the HCPTEs by cross‐referencing the HCPTE annotation file with the gene annotation file, using the bedtools suite. We used the “closest” program (‐D b ‐fu ‐io; b being the gene annotation file) to identify the closest (but not intersecting) gene downstream each HCPTE. We only retained the entries with a maximum distance of 1 kb between the HCPTE and gene boundaries. We identified the insertions in the gene using the “intersect” tool (‐wo).

Then, we performed a manual bioinformatics functional analysis for each gene potentially impacted by HCPTEs. Protein sequences were extracted from the *M*. *incognita* predicted proteome (Blanc‐Mathieu et al., 2017) and blasted (blastp; default parameters) against the non‐redundant protein sequence database (NR) from the NCBI (https://blast.ncbi.nlm.nih.gov/). The same sequences were also used on the InterProScan website (https://www.ebi.ac.uk/interpro/) to perform an extensive search on all the available libraries of conserved protein domains and motifs.

Then, for each gene potentially impacted by HCPTEs, we performed an orthology search on the WormBase Parasite website (https://parasite.wormbase.org/) using gene accession numbers and the precomputed ENSEMBL Compara orthology prediction (Herrero et al., 2016).

Finally, we analyzed the expression levels of the genes potentially impacted by HCPTEs extracting the information from the RNA‐seq analysis of four *M*. *incognita* life stages performed previously (see Putative transposition machinery identification section).

#### 2.5.3 Experimental validation of HCPTE loci

To experimentally validate in silico predictions of TE neo‐insertions with potential functional impact, we selected five candidates among the HCPTE loci and performed a PCR experiment. To run this experiment, we used DNA remaining from extractions performed on the *M. incognita* isolates for a previous population genomics analysis (Koutsovoulos, Marques et al., 2020). We selected loci to be validated based on the following criteria:

● The predicted insertion must be in a genic or potential regulatory region (max 1 kb upstream of a gene) as the most evident criterion for a potential functional impact.
● The element must be short enough (2.5 kb max) to be amplified by PCR and SANGER‐sequenced using standard techniques and material.
● To validate the predicted impacted gene actually exists, it must be supported by substantial expression data in the reference isolate Morelos.
● To maximize the chances the genes have effects on biological traits characteristic of the root‐knot nematodes, the impacted gene must be *Meloidogyne*‐specific.

Once all these criteria were applied, we maximized the diversity of TE orders involved and this resulted in the 5 loci presented in Results section.

##### Primer design and PCR amplification

We designed primers for the PCR analysis using the Primer3Plus web interface (Untergasser et al., 2007). The set of 10 primers with the corresponding sequences and expected amplicon sizes with, or without TE insertion, is shown in Table S2 and Kozlowski, Hassanaly‐Goulamhoussen et al. (2020). We used primers amplifying the whole actin‐encoding gene (Minc3s00960g19311) as positive control.

PCR experiments were performed on *M*. *incognita* Morelos isolate and 11 Brazilian isolates: R1‐2, R1‐3, R1‐6, R2‐1, R2‐6, R3‐1, R3‐2, R3‐4, R4‐1, R4‐3, and R4‐4.

R3‐1 presented no amplification in any of the tested loci nor the positive control (actin) and was thus discarded from this analysis.

PCR mixture contained 0.5 µmol of each primer, 1x MyTaq™ reaction buffer and 1.0 U of MyTaq™ DNA polymerase (Bioline Meridian Bioscience) adjusted to a total volume of 20 µl. PCR amplification was performed with a TurboCycler 2 (Blue‐Ray Biotech Corp.). PCR conditions were as follows: initial denaturation at 95°C for 5 min, followed by 35 cycles of 95°C for 30 s, 56°C for 30 s of annealing, and 72°C for 3 min of extension, and the program ends with a final extension at 72°C for 10 min. Aliquots of 5 µl were migrated by electrophoresis on a 1% agarose gel (Sigma Chemical Co.) for 70 min at 100 V. The size marker used is 1 kb Plus DNA Ladder (New England Biolabs Inc.), containing the following size fragments in bp: 100, 200, 300, 400, 500, 600, 700, 900, 1000, 1200, 1500, 2000, 3000, 4000, 5000, 6000, 8000, and 10,000.

##### Purification and sequencing of PCR amplicons

Amplicon bands were revealed using ethidium bromide and exposure to ultraviolet radiation. PCR product bands were excised from the agarose gel with a scalpel and purified using MinElute Gel Extraction Kit (Qiagen) before sequencing, following the manufacturer's protocol. PCR products were sequenced by Sanger sequencing (Eurofins Genomics).

Forward (F) and reverse (R) sequences were blasted individually (https://blast.ncbi.nlm.nih.gov/; optimized for “somewhat similar sequences,” default parameters) to the expected TE consensus sequence and to the genomic region surrounding the predicted insertion point (2 kb region: 1 kb upstream the predicted insertion point and 1 kb downstream). When no significant hit was found, the sequence was blasted against the *Meloidogyne* reference genomes available at (https://meloidogyne.inrae.fr/), the whole TE consensus library, and the NR database on the NCBI blast website.

## 3 RESULTS

### 3.1 The *Meloidogyne incognita* TE landscape is diverse but mostly composed of DNA transposons

**FIGURE 1:**
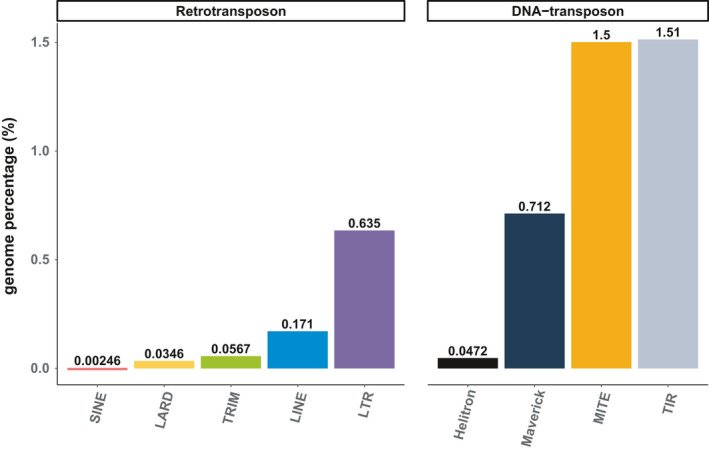
Canonical TE annotation distribution in *Meloidogyne* *incognita* genome. Genome percentage is based on a *M*. *incognita* genome size of 183,531,997 bp (Blanc‐Mathieu et al., 2017). More detailed statistics are available in Table S4

We used the REPET pipeline (Flutre et al., 2011; Quesneville et al., 2005) to predict and annotate the *M*. *incognita* repeatome (see Methods). Here, we define the repeatome as all the repeated sequences in the genome, excluding simple sequence repeats (SSR or microsatellites). The repeatome spans 26.38% of the *M*. *incognita* genome length (Table S3). As we wanted to assess whether TE movements contributed to genomic plasticity, we applied a series of stringent filters on the whole repeatome to retain only repetitive elements harboring the characteristic signatures of known retro and DNA transposon orders, hereafter denominated “canonical” TEs (see Methods and Kozlowski, 2020a). We identified 480 different TE consensus sequences that allowed annotation of 9633 canonical TEs, spanning 4.67% of the genome (Table S4). Both retro (Class I) and DNA (Class II) transposons (Wicker et al., 2007) compose the *M*. *incognita* TE landscape with 5 of 7 and 4 of 5 of the known TE orders represented, respectively, showing a great diversity of elements (Figure 1). Canonical retrotransposons and DNA transposons respectively cover 0.90 and 3.77% of the genome. Terminal inverted repeat (TIR) and miniature inverted repeat transposable element (MITE) DNA transposons alone represent almost two‐thirds of the *M*. *incognita* canonical TE content (64.49%). Hence, the *M*. *incognita* TE landscape is diverse but mostly composed of DNA transposons.

As a technical validation of our repeatome annotation protocol (see Methods, Figure S2), we performed the same analysis in *C*. *elegans*, using the PRJNA13758 assembly (The C. elegans Genome Sequencing Consortium, 1998). We compared our results (Kozlowski, 2020b) with the reference report of the TE landscape in this model nematode (Bessereau, 2006). We estimated that the *C*. *elegans* repeatome spans 11.81% of its genome (Table S5), which is close to the 12% described in Bessereau (2006). The same resource also reported that MITEs and LTR respectively compose ~2% and 0.4% of the *C*. *elegans* genomes, while we predicted 1.8% and 0.2%. Predictions obtained using our protocol are thus in the range of previous predictions for *C*. *elegans*, which suggest our repeatome prediction and annotation protocol are accurate.

The WormBook resource (Bessereau, 2006) mentioned that most of *C*. *elegans* TE sequences "are fossil remnants that are no longer mobile" and that active TEs are DNA transposons. This suggests a stringent filtering process is necessary to isolate TEs that are the most likely to be active (e.g., the “canonical” ones). Using the same postprocessing protocol as for *M*. *incognita*, we estimated that canonical TEs span 3.60% of the *C*. *elegans* genome, with DNA transposon alone representing 76.6% of these annotations (Figure S4; Table S6).

### 3.2 Canonical TE annotations show high similarity to their consensus sequences, and some present evidence for transposition machinery

**FIGURE 2:**
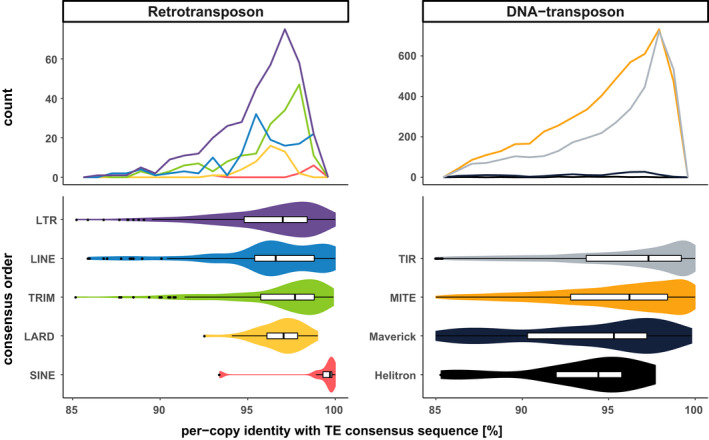
Per‐copy identity rate with consensus. Top frequency plots show the distribution of TE copies count per order in function of the identity % they share with their consensus sequence. To facilitate interorder comparison, bottom violin plots display the same information as a density curve, but also encompass boxplots. Each color is specific to a TE order

Canonical TE annotations have a median nucleotide identity of 97% with their respective consensus sequences, but the distribution of identity values varies between TE orders (Figure 2; Table S7). Most of the TEs within an order share a high identity level with their consensuses, the lowest values being observed for Helitron and Maverick elements. Yet, more than half of these elements share above 94% identity with their consensuses (Figure S5). Although it might be hypothesized the lower identities would be due to bigger length (Figure S6), we showed no evident correlation between the % identity copies share with their consensus and the proportion of consensus length covered (Figure S5). Even considering our inclusion threshold at minimum 85% identity (see Methods), the overall distribution of average % identities tends to be asymmetrical and skewed toward higher values (Figure 2).

Among DNA transposons, identity profiles of MITEs and TIRs to their consensuses were the most shifted to high values; one fourth of the TIR annotations share above 99% identity with their consensus (Figure 2; Tables S4 and S7).

Among retrotransposon, SINEs (present in very low numbers) and TRIMs show similar profiles with a quite narrow peak at more than 97% identity. Overall, these results indicate that notwithstanding small differences between orders, the canonical TEs show a high similarity with their consensuses.

High identity of TE annotations to their consensus can be considered a proxy of their recent activity (Bast et al., 2015; Lerat et al., 2019). To further investigate whether some TEs might be (or have been recently) active, we searched for the presence of genes involved in the transposition machinery within *M*. *incognita* canonical TEs (see Methods). Among the canonical TE annotations, 6.21% (598/9633) contain at least one predicted protein‐coding gene, with a total of 893 genes involved. Of these 893 genes, 344 code for proteins with at least one conserved domain known to be related to transposition machinery. We found that 31.98% (110/344) of the transposition machinery genes had substantial expression support from RNA‐seq data. In total, 106 canonical TE annotations contain at least one substantially expressed transposition machinery gene (Kozlowski, Da Rocha et al., 2020). These 106 TE annotations correspond to 39 different TE consensuses, and as expected, only consensuses from the autonomous TE orders, for example, LTRs, LINEs, TIRs, Helitron, and Maverick, present TE copies with substantially expressed genes coding for transposition machinery (Table S8). Conversely, the non‐autonomous TEs do not contain any transposition machinery gene at all. This suggests that some of the detected TEs have functional transposition machinery, which in turn could be hijacked by the non‐autonomous elements.

Overall, the presence of a substantial proportion of TE annotations highly similar to their consensuses combined with the presence of genes coding for the transposition machinery and supported by expression data suggests some TE might be active in the genome of *M*. *incognita*.

### 3.3 Thousands of loci show variations in TE presence frequencies across *Meloidogyne incognita* isolates

We used the PoPoolationTE2 pipeline (Kofler et al., 2016) on the *M*. *incognita* reference genome (Blanc‐Mathieu et al., 2017) and the canonical TE annotation to detect variations in TE frequencies across the genome between 12 geographical isolates (see Methods; Kozlowski, 2020b; Figure S2). The Morelos isolate from Mexico was the one used to produce the *M*. *incognita* reference genome (Blanc‐Mathieu et al., 2017). The 11 other isolates come from different locations across Brazil, and present four different ranges of compatible hosts (referred to as R1, R2, R3, and R4; see Figure S1) and currently infected crop species (Koutsovoulos, Marques et al., 2020). Each isolate was reared from the offspring of a single female, and approximately 1 million individuals per isolate were pooled to gather enough material for DNA extraction and pool‐seq paired‐end Illumina sequencing. For each locus, each isolate has an associated frequency value representing the proportion of reads in the pool supporting the presence of a TE at this location.

We identified 3514 loci where the amplitude of frequency variation between at least two isolates was above our estimated PoPoolationTE2 error rate (0.00972, i.e., less than 1%; see Methods), and thus likely to represent a biological reality.

**FIGURE 3:**
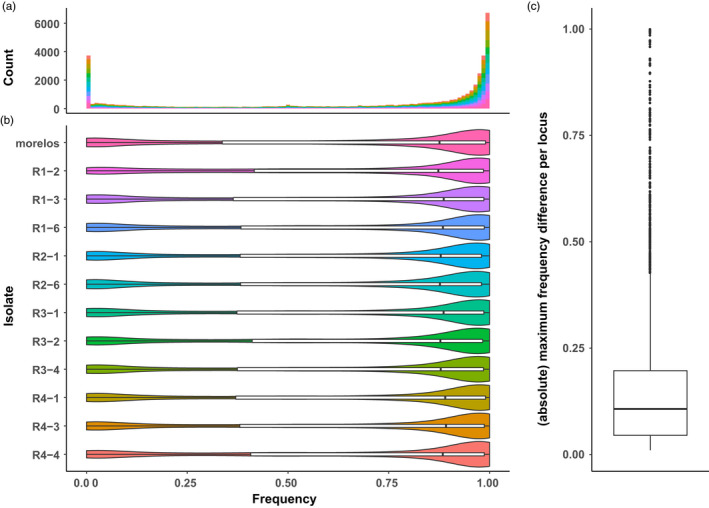
TE frequency distribution. The histogram (a) and violin plot (b) represent the TE frequency distribution per isolate. The color chart is identical between the two figures. Both representations reveal that in all the isolates, only a few TE are found with intermediate frequencies. Right boxplot (c) represents the frequency absolute maximum difference per locus. For a given locus, it illustrates the frequency variability between isolates. The higher is the value, the more important is the frequency difference between at least two isolates. A value of 1 implies that the TE is absent in at least one isolate, while it is present in 100% of the individuals of at least another isolate

Overall, the distribution of within‐isolate frequencies is bimodal (Figure 3a), and this pattern is common to all the isolates, including the reference Morelos isolate (Figure 3b). On average, 21.1% of the loci have within‐isolate frequencies <25%, 60.7% have frequencies >75%, and only 18.2% show intermediate frequencies. Hence, most of the within‐isolate TE frequencies pack around extreme values, for example, <25% or >75%, which makes sense with the supposed clonal reproduction. Indeed, given the reproductive mode and because each isolate was reared from the offspring of one single female, initial frequencies are expected to be either 0 or 1 depending on whether the TE was respectively present or absent at a given locus in the progenitor female. Variations around these initial extreme frequencies might be due to the tendency of PoPoolationTE2 to underestimate high frequencies and to overestimate low frequencies. However, true within‐isolate variations in the presence/absence of a TE at a given locus have been experimentally confirmed via PCR experiments in another complementary study (Kozlowski, 2020c). Moderate variations around these extreme values could be due to progressive vanishing/fixation of a TE at a given locus within the isolate generation after generation. Intermediate frequencies (e.g., 0.25–0.75) are less expected and can hardly be explained by this phenomenon alone. We hypothesize that the bottlenecks applied when a subsample of the total population is extracted for genome sequencing can alter the TE frequency distribution and contribute to the relatively rare within‐isolate intermediate frequencies observed.

Nevertheless, these statistics provide no information about the frequency variability between isolates for a given locus, which are the ones corresponding to differences in TE presence support at a genomic locus across isolates. To address this question, for each locus, we computed the absolute maximum frequency difference between isolates (Figure 3c). We found that the maximum frequency variation across the isolates is smaller than 20% in 75% of the loci (2634/3514). Hence, most of the loci show little‐to‐moderate variations in frequencies between isolates. Combined with the previous result, this implies that for most loci, TEs are present either at a high or at a low frequency among all isolates. However, some TE loci show more contrasted variations and will be the focus of our further studies.

### 3.4 Variations in TE frequencies across isolates recapitulate their divergence at the sequence level

We performed a neighbor‐joining phylogenetic analysis of *M*. *incognita* isolates based on a distance matrix constructed from TE frequencies (3514 loci; see methods). We also performed a maximum‐likelihood (ML) analysis based on SNV in coding regions as previously identified in Koutsovoulos, Marques et al. (2020) adding the reference isolate Morelos.

**FIGURE 4:**
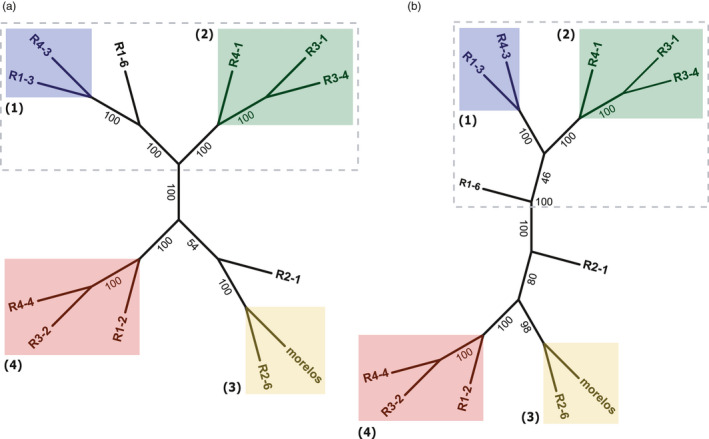
Phylogenetic tree for *Meloidogyne* *incognita* isolates. (a) Maximum‐likelihood (ML) phylogenetic tree based on SNV present in coding sequences. Branch length not displayed (see Figure S7 for a version with branch length displayed). (b) Neighbor‐joining (NJ) phylogenetic tree based on TE frequency Euclidean distances between isolates. Branch length not displayed (see Figure S7 for a version with branch length displayed). In both trees, bootstrap support values are indicated on the branches. Isolates enclosed in the dashed area form a super‐cluster composed of the clusters (1) and (2), and the isolate R1‐6

As shown in Figure 4, the TE‐based and SNV‐based tree topologies are highly similar. In particular, the two trees allowed defining four highly supported clades, with bootstrap values ≥98. The four clades were identical, including branching orders for clades 2 and 4 (the two other clades containing each only two isolates). R1‐6 and R2‐1 positions slightly differed between the SNV‐based (A) and TE‐based (B) trees. However, in both trees R1‐6 is more closely related to clusters 1 and 2 than the rest of the isolates, and similar observations can be drawn for R2‐1 with clusters 3 and 4.

Altogether, the similarity between the SNV‐based and TE frequency‐based trees indicates that most of the phylogenetic signal coming from variations in TE frequencies between isolates recapitulates the SNV‐based genomic divergence between isolates and thus genome diversification within the species.

### 3.5 Although most TE loci are stable, some show substantial variations including differential insertions/deletions among isolates

**FIGURE 5:**
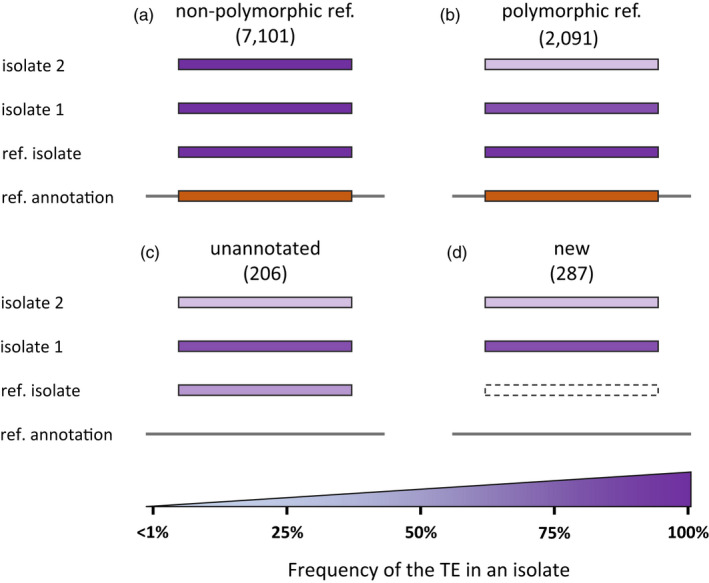
Categories of polymorphic TE loci. Orange boxes illustrate the presence of a TE at this locus in the reference genome annotation. Purple boxes illustrate the percentage of individuals in the isolates for which the TE is present at this locus (i.e., frequency). Frequency values are reported as color gradients. (a) Nonpolymorphic ref. TE locus: a TE is predicted in the reference annotation (orange box) AND no frequency variation exceeding 1% between isolates (Morelos included) is detected. (b) Polymorphic ref. locus: a TE is predicted in the reference annotation, is detected in the reference isolate Morelos with a frequency >75%, and the presence frequency varies (>1%) in at least one isolate. (c) unannotated loci: no TE was predicted at this locus in the reference annotation but one is detected at a frequency >25% in the reference isolate Morelos, and possibly in other isolates. (d) New TE loci: No TE was predicted at this locus in the reference genome annotation and none is detected in the reference isolate (dashed box, frequency <1%), but a TE is detected in at least another isolate with a frequency ≥25%

As explained below (see also Methods and Figures S2 and S3), we categorized all the loci with TE frequency variations between the isolates by (i) comparing their position with the TE annotation in the reference genome, (ii) analyzing TE frequency in the reference isolate Morelos, and (iii) comparing TE frequencies detected for each isolate to the reference isolate Morelos. This allowed defining, on the one hand, nonpolymorphic and hence stable reference annotation, and, on the other hand, three categories of polymorphic (variable) loci (Figure 5).

**FIGURE 6:**
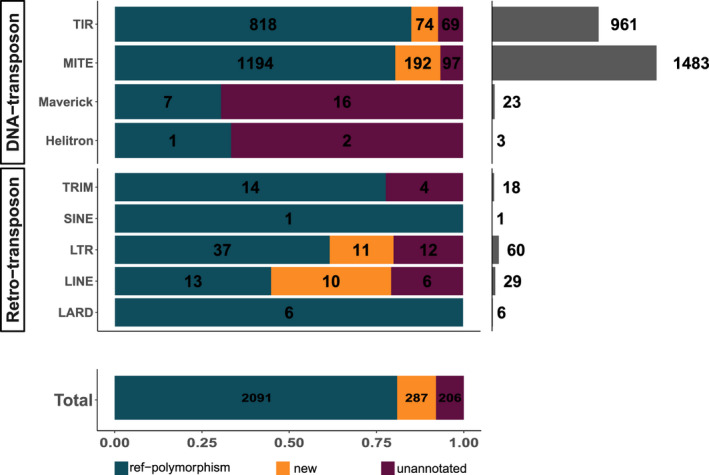
TE polymorphism count per orders and types. The top left bar plot shows TE polymorphisms distribution per type and per order. The bottom‐left bar plot summarizes TE polymorphisms distribution per type. In both bar plots, the values in black represent the count per polymorphism type. The top‐right bar plot illustrates the total number of polymorphisms per order

Overall, 73.5% (2584/3514) of the loci with TE frequency variations could be assigned to one of the three categories of TE polymorphisms (b, c, d in Figure 5) and the decomposition per TE order is given in Figure 6 and Table S9. Most polymorphic loci (80.92%; 2091/2584) correspond to an already existing TE annotation in the reference genome whose presence is confirmed (frequency >75%) at least in the reference isolate Morelos but varies in at least another isolate. Note that the ancestral state being unknown, extreme cases of variations in these polymorphic loci could equally represent a TE insertion at least in the reference strain Morelos or a loss in one or several other isolates. These polymorphic loci encompass ~21.6% (2091/9702) of the canonical TE annotations, in total. These loci will be referred to as “polymorphic reference loci” from now on (Figure 5b), and they concern both retro and DNA transposons.

Then, we considered as “new TE loci”, TEs present at a frequency >25% in at least one isolate at a locus where no TE was annotated in the reference genome and the frequency of TE presence was lower than the estimated error rate (~1%) in the reference Morelos isolate (Figure 5d). In total, 11.11% (287/2584) of the detected TE polymorphisms correspond to such new TE loci. It should be noted here that these new loci can equally represent TE loss in Morelos or TE insertion in at least another isolate. Comparison with the phylogenetic pattern of presence/absence will allow distinguishing further the most parsimonious of these two possibilities (see next sections).

Finally, we classified as “unannotated loci” (Figure 5c) (7.97%; 206/2584) the loci where no TE was initially annotated by REPET in the reference genome, but a TE was detected at a frequency >25% at least in the ref isolate Morelos by PoPoolationTE2. It should be noted that 58.73% (121/206) of these loci correspond to draft annotations that have been discarded during the filtering process to only select the canonical annotations. These draft annotations might represent truncated or diverged versions of TE that exist in a more canonical version in another locus in the genome. Half of the remaining “unannotated loci” (42/85) are detected with low‐to‐moderate frequency (<42.6%) in the reference isolate Morelos. We hypothesize that because they represent the minority form, these regions were not taken into account during the assembly of the genome. This would explain why these TEs could not be detected in the genome assembly by REPET (assembly‐based approach) but were identified with a read‐mapping approach on the genome complemented by the repeatome by PoPoolationTE2. The remaining “unannotated loci” might correspond to REPET false negatives, PoPoolationTE false positives, or a combination of the two. Nonetheless, we can notice these cases only represent 1.63% (42/2584) of the detected polymorphic TEs.

### 3.6 TIR and MITE elements are over‐represented among TE polymorphisms

By themselves, MITE and TIR elements encompass 94.58% (2444/2584) of the categorized TE polymorphisms (Figure 6).

We showed that the polymorphism distribution varies significantly between the four categories presented in Figure 5 (chi‐square test, *p*‐value <2.2e‐16), indicating that some TE orders are characterized by specific polymorphism types.

The analysis of the chi‐square residuals (Figure S8) shows MITEs and TIRs are the only orders presenting a relative lack of nonpolymorphic “stable” TEs. Hence, these two TE orders are significantly enriched among polymorphic loci despite their higher abundance in the genome. MITEs are over‐represented in both TE polymorphism types (polymorphic ref. loci and new TE loci; Figure 5b,d), suggesting a variety of activities within this order. On the other hand, TIRs are found in excess in ref‐polymorphisms but lack in new TE loci. This lack of new TIR loci may indicate a recent lower activity in this order or a more efficient negative selection.

Finally, we observed a strong excess of Mavericks among the unannotated loci as almost 70% of Maverick polymorphisms (16/23) (Figure 6) fell into this category. Consistent with the observation that, globally, >50% of the unannotated loci were actually draft TE predictions eliminated afterward during filtering steps, ¾ (12/16) of the Maverick elements were also present in the draft annotations but later eliminated during filtering steps.

Overall, in proportion, MITE and TIR elements are significantly over‐represented in TE polymorphisms, indicating their frequencies at loci show more variations between isolates than the other TE orders.

### 3.7 Some polymorphic loci with contrasted frequency variations between isolates most probably represent TE neo‐insertions

We investigated the variability of TE presence frequency per locus between the 12 isolates for all the categorized polymorphic loci in the genome.

In ~3/4 (1911/2584) of the categorized polymorphic TE loci, although variations in presence frequency between isolates were above the estimated error rate (<1%), they remained at relatively low amplitude (maximum frequency variation between isolates ≤25% for a given locus) (see Methods; Figure S3). Most of these cases (97.95%; 1872/1911) concern loci where the TE is present at a high frequency in all isolates (>75%). These loci might be considered as conserved and relatively stable in all the isolates. In the remaining 2.04% (39/1911), the TE frequency is either between 25 and 50% or between 50 and 75% in all isolates. Consistent with our methodology, all the high‐frequency loci correspond to ref‐polymorphisms, while all the intermediate frequency loci belong to unannotated loci.

In contrast, 673 polymorphic TE loci showed higher amplitudes of variations in TE presence frequency (>25%) between at least two isolates (median difference = 31.35%). Among the most extreme cases of frequency variation per locus, we identified 33 loci in which the TE is found with high frequencies (>75%) for some isolate(s), while it is absent or rare (frequency <25%) in the other(s). These loci will be from now on referred to as HCP standing for "highly contrasted polymorphic” TE loci. Because they are highly contrasted, these loci might represent fixed differential TE insertions or deletions across isolates and will be the focus of the following analyses.

HCP TE loci encompass 19 MITE elements, 12 TIRs, and 2 LINEs (Table S10). We can also notice that some consensuses are more involved in HCP TE loci as two TE consensuses alone are responsible for 72.72% (18/33) of these polymorphisms (one MITE consensus involved in 10 HCP loci and one TIR consensus involved in eight such loci).

**FIGURE 7:**
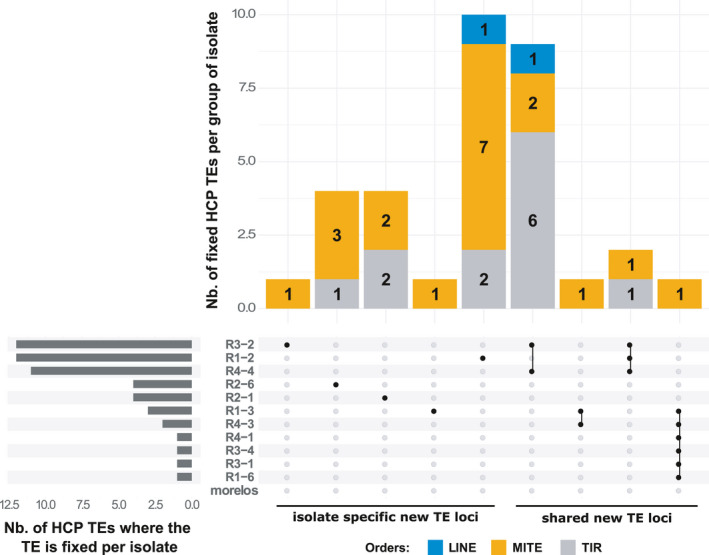
Distribution of the 33 HCP new TE loci across isolates. The central plot shows how many and which isolate(s) share common highly contrasted polymorphic (HCP) new TE loci, every line representing an isolate. Columns with several dots linked by a line indicate shared HCP new TE loci between isolates. Each dot represents which isolate is involved. Columns with a single dot design isolate‐specific HCP new TE loci. The top bar plot indicates how many HCP new TE loci the corresponding group of isolate shares. The left‐side bar plot specifies how many HCP new TE loci are found in a given isolate

Interestingly, all the HCP TE loci correspond to new TE loci regarding the reference genome, meaning that no TE was annotated in the reference genome at this location and the TE presence frequency is <1% in the Morelos reference isolate. As described in Figure 7, most of these fixed new TE loci (20/33) are specific to an isolate and most probably represent isolate‐specific neo‐insertions conserved in the offspring rather than multiple independent losses.

However, we also found new TE loci shared by two (10/33), three (2/33), or even six isolates (1/33). Interestingly, all the shared new TE loci were between isolates present in a same cluster in the phylogenetic trees (TE‐based and SNV‐based in Figure 4), suggesting they have been inherited by a common ancestor, then maintained at high frequency in the descending isolates. For example, two new TE loci are shared by isolates R4‐4, R1‐2, and R3‐2, which belong to the same cluster 1, and one new TE locus is shared by isolates R4‐3 and R1‐3, which belong to the same cluster 2.

Hence, the phylogenetic distribution reinforces the idea that these new TE loci are more likely to represent branch‐specific neo‐insertions than multiple independent losses, including in the reference isolate Morelos. Even the new TE locus shared by 6 isolates follows this pattern as all the concerned isolates belong to the same super‐cluster composed of the cluster 2 and 3 plus isolate R1‐6 (dashed line in Figure 4). However, in this case a neo‐insertion in the common ancestor of these isolates is equally parsimonious than a deletion in the ancestor of all the other isolates.

Isolates R1‐2, R3‐2, and R4‐4 show the highest number of new TE loci. However, their profiles are quite different. In R1‐2, 10/12 HCP TE loci are isolate‐specific and thus likely represent neo‐insertions, while most of the HCP TE loci involving R3‐2 and R4‐4 are new loci shared with closely related isolates and thus probably inherited from a common ancestor. This is also consistent with the topology and branch lengths of the SNV‐based and TE‐based phylogenies (Figure S7), which shows that R1‐2 is the most divergent isolate with the longest branch length, while R3‐2 is quite close to R4‐4 and has a relatively short branch.

### 3.8 Functional impact of TE neo‐insertion and validation of in silico predictions

Interestingly, two‐thirds (22/33) of the HCP new TE loci correspond to insertions inside a gene or in a possible regulatory region (1‐kb region upstream of a gene). These insertions maintained at high frequency might have a functional impact in *M*. *incognita*. Overall, 27 different genes (26 coding for proteins and one tRNA gene) are possibly impacted by the 22 insertions, some genes being in the opposite direction at an insertion point (overlapping this insertion point or being at max 1 kb downstream). More than 80% of these genes (22/27) show a substantial expression level during at least one life stage of the nematode life cycle (in the Morelos isolate), suggesting the impacted genes are functional in the *M*. *incognita* genome (see Methods). Some of the impacted genes (40.74%, 11/27) are specific to the *Meloidogyne* genus (they have no predicted orthologs in other nematodes, according to WormBase Parasite). Ten of these *Meloidogyne*‐specific genes are widely conserved in multiple *Meloidogyne* species, reinforcing their possible importance in the genus, and one is so far only present in *M*. *incognita*. Interestingly, further similarity search using BLASTp against the NCBI’s nr library returned no significant hits, suggesting these proteins are so far *Meloidogyne*‐specific and do not originate from horizontal gene transfers of non‐nematode origin. Among the remaining genes, one is present in multiple *Meloidogyne* species and otherwise only found in other plant parasitic nematode (PPN) species (*Ditylenchus destructor*, *Globodera rostochiensis*) (Table S11). Conservation of these genes across multiple PPN but exclusion from the rest of the nematodes or other species suggests these genes might be involved in important functions relative to these organisms lifestyle, including plant parasitism itself.

To experimentally validate in silico predictions of TE insertions with potential functional impact, we performed PCR experiments on 5 of the 22 HCP new TE loci falling in coding or possible regulatory regions (see Methods for selection criteria). To perform these PCR validations, we used the DNA remaining from previous extractions performed on the *M*. *incognita* isolates for population genomic analysis (Koutsovoulos, Marques et al., 2020). Basically, the principle was to validate whether the highly contrasted frequencies (>75%/<25%) obtained by PoPoolationTE2 actually corresponded to absence/presence of a TE at the locus under consideration (see Methods). One isolate (R3‐1) presented no amplification in any of the tested loci nor in the positive control. After testing the DNA concentration in the sample, we concluded that the DNA quantity was too low in this isolate and decided to discard it from the analysis.

**FIGURE 8:**
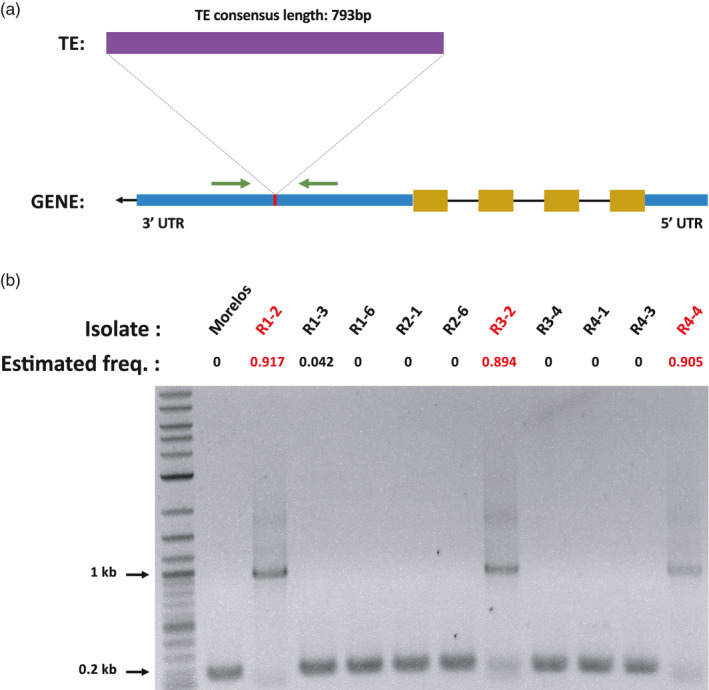
Experimental validation of a predicted TE insertion. (a) Diagram of the TE insertion. The insertion of the MITE element occurs in the 3′UTR region of the gene (Minc3s00026g01668). Blue boxes illustrate the 3′ and 5′ UTR regions of the gene, while the yellow boxes picture the exons. Green arrows represent the primers used to amplify the region. Gene subparts and TE representations are not at scale. Predicted size of the amplicon: 973 bp with the TE insertion, 180 bp without. (b) PCR validation of the TE insertion. Estimated freq. values correspond to the proportion of individuals per isolate predicted to have the TE at this position (PoPoolationTE2). Isolates in red were predicted to have the TE inserted at this locus. Only these isolates show an amplicon with a size suggesting an insertion (sequences are available in Kozlowski, Hassanaly‐Goulamhoussen et al., 2020)

For four of the five tested HCP new TE loci, we could validate by PCR the in silico predicted differential presence/absence of a sequence at this position, across the different isolates (Figure 8; Kozlowski, Hassanaly‐Goulamhoussen et al., 2020).

In one of the five tested loci, named locus 1, we could (i) validate by PCR the presence of a sequence at this position for the isolates presenting a PoPoolationTE2 frequency >75% and absence for those having a frequency <25%; and (ii) also validate by sequencing that the sequence itself corresponded to the TE under consideration (a MITE). This case is further explained in detail below and in Figure 8.

The PoPoolationTE2‐estimated frequencies are higher than 75% in three isolates (R1‐2, R3‐2, and R4‐4) derived from a common ancestor (cluster 4 in Figure 4) for one MITE. Thus, this MITE was probably inserted in this common ancestor and maintained at high frequency in the three descending isolates. We assumed the TE is absent from the rest of the isolates as all of them display frequencies <5%. To validate this differential presence across the isolates, we designed specific primers from each side of the estimated insertion point so that the amplicon should measure 973 bp with the TE insertion and 180 bp without.

The PCR results are consistent with the frequency predictions as only R1‐2, R3‐2, and R4‐4 display a ~1 kb amplicon, while all the other isolates show a ~0.2 kb amplicon (Figure 8). Hence, as expected, only the three isolates with a predicted TE frequency >75% at this locus exhibit a longer region, compatible with the MITE insertion.

To validate the amplified regions corresponded to the expected MITE, we sequenced the amplicons for the three isolates and aligned the sequences to the TE consensus and the genomic region surrounding the estimated insertion point (Kozlowski, Hassanaly‐Goulamhoussen et al., 2020). Amplicon sequences of the three isolates covered a significant part of the TE consensus sequence length (>78%) with high identity (>87%) and only a few gaps (<5%). These results confirm that the inserted sequence corresponds to the predicted TE consensus. Moreover, the three amplicons aligned on the genomic region downstream of the insertion point with high identity (≥99%), which helped us further determine the real position of the insertion point. The real insertion point is 26 bp upstream of the one predicted by PoPoolationTE2 and falls in the forward primer sequence. This explains why the amplicon sequences do not align on the region upstream of the insertion point.

We also noticed that the inserted TE sequences slightly diverged between the isolates, while the genomic region surrounding the insertion point remains identical. Interestingly, the level of divergence in the TE sequence does not follow the phylogeny as R‐4_4 is closer to R‐1_2 than to R‐3_2 (Table S12).

Finally, in the Morelos, R‐2_1, and R‐2_6 isolates, the sequencing of the amplicon validated the absence of insertions. Indeed, the sequences aligned on the genomic region surrounding the insertion point with high identity (99, 97, and 87%, respectively) but not with the MITE consensus.

Hence, we fully validated experimentally the presence/absence profile across isolates predicted in silico at this locus.

In the *M*. *incognita* genome, this MITE insertion is predicted to occur in the 3′ UTR region of a gene (Minc3s00026g01668). This gene has no obvious predicted function, as no conserved protein domain is detected and no homology to another protein with an annotated function could be found. However, the gene model is supported by expression data during the whole life cycle of the nematode (Kozlowski, Da Rocha et al., 2020) and orthologs were found in the genomes of several other *Meloidogyne* species (*M*. *arenaria*, *M. javanica*, *M. floridensis*, *M. enterolobii*, and *M. graminicola*), ruling out the possibility that this gene results from a prediction error from gene calling software. The expression and broad conservation of this gene in the *Meloidogyne* genus suggests this gene might be important for *Meloidogyne* biology and survival.

Consequently, the insertion of a MITE in R‐1_2, R‐3_2, and R‐4_4 genomes at this locus could have functional impacts.

## 4 DISCUSSION

### 4.1 TE landscape in nematode genomes and possible recent activity in *Meloidogyne incognita*

In this analysis, we have annotated TEs in the genome of *M*. *incognita* and used variations in TE frequencies between geographical isolates across loci as a reporter of their activity. The *M*. *incognita* TE landscape is more abundant in DNA than in retrotransposons, and using the same methodology, we confirmed a similar trend in the genome of *C*. *elegans*, despite the *Caenorhabditis* and *Meloidogyne* genus being separated by >200 millions of years of evolution (Kumar et al., 2017). Interestingly, even if the methodology used was different, a similar observation was made at the whole *Nematoda* level (Szitenberg et al., 2016), suggesting a higher abundance of DNA transposons might be a general feature of nematode genomes.

We have shown 75% of the polymorphic TE loci in *M*. *incognita* display moderate‐frequency variations between isolates (<25%), a majority being found with high frequencies (>75%) in all the isolates simultaneously. Hence, a substantial part of the TE can be considered as stable and fixed among the isolates.

Nevertheless, the remaining quarter of polymorphic TE loci present frequency variations across the isolates exceeding 25%. This observation concerns both the TE already present in the reference genome and new TE loci. We even detected loci where the TE frequencies were so contrasted between the isolates (HCP TE loci) that we could predict the TE presence/absence pattern among the isolates. Such frequency variations between isolates, and the fact that most of the HCP loci represent lineage or isolate‐specific TE insertions, constitute strong evidence for TE activity in the *M*. *incognita* genome.

In *C*. *elegans*, multiple TE families have also shown a substantial level of activity across different populations (Laricchia et al., 2017). However, this analysis was based on binary presence/absence data of TE at loci across populations and thus neither provided information about the amplitude of TE frequency variations between nor within isolates. At this stage, it is thus impossible to compare the within‐ and between‐isolate variability in TE frequencies we observed in *M*. *incognita* to other nematodes.

It should be noted here that the total TE activity in the *M*. *incognita* genome is probably underestimated, in part because of the stringent filters we applied to eliminate false positives as much as possible, and in another part because of the intrinsic limitations of the tools, such as the incapacity of PoPoolationTE2 to detect nested TEs (Kofler et al., 2016).

We then evaluated how recent this activity could be, using % identity of the TE copies with their respective consensuses as a proxy for their age as previously proposed in other studies (Bast et al., 2015; Lerat et al., 2019). We showed that a substantial proportion of the canonical TE annotations were highly similar to their consensus, indicating most of these TE copies were recent in the genome. The probable recent hybrid origin of *M*. *incognita* (Blanc‐Mathieu et al., 2017) is consistent with a recent TE burst in the genome. Indeed, as further explained in the last section of the discussion, it is well established that hybridization events can lead to a relaxation of the TE silencing mechanisms and consequently to a TE expansion (Belyayev, 2014; Guerreiro, 2014; Rodriguez & Arkhipova, 2018).

However, as suggested in Bourgeois and Boissinot (2019), the extent of this phenomenon might differ depending on the TE order. In *M. incognita*, MITEs and TIRs alone account for ~2/3 of the canonical TE annotations, but their fate in the genome seems to have followed different paths. Indeed, as illustrated in Figure 2, MITEs show a wide range of identity rates with their consensus, which suggests they might have progressively invaded the genome being uncontrolled or poorly controlled as suggested for the rice genome (Lu et al., 2017). On the opposite, almost all the TIR copies share high percentage identity with their consensuses, which could be reminiscent of a rapid and recent burst. Nevertheless, this burst could have quickly been under control as, according to chi‐square residuals (Figure S8), new TIR loci are significantly less numerous than expected owing to their abundance in the genome. These differences between the distribution of MITE and TIR identities to their consensuses are possibly linked to differences in the TE length itself. Indeed, TIRs are usually expected to be at least twice longer than MITEs and their accumulation may be subject to higher counter‐selection than MITEs as length‐dependent selection on TE persistence has previously been observed in different animals (Bourgeois & Boissinot, 2019). Interestingly, in *C*. *elegans*, the Tc1/Mariner TIR DNA element was shown to be the most active while, so far, no evidence for active retrotransposition was shown in this species (Bessereau, 2006; Laricchia et al., 2017).

Because no molecular clock is available for *M*. *incognita*, it is impossible to evaluate more precisely when TE bursts would have happened and how fast each TE from each order would have spread in the genome. Such bursts can be very recent, including in animal genomes as exemplified by the P‐element, which invaded the genome of some Drosophila populations in just 40 years (Anxolabéhère et al., 1988). While an absolute dating of TE activities in *M*. *incognita* is currently not possible, a relative timing of the events regarding population diversification can still be deduced from the distribution of TE locus frequencies across isolates. Indeed, we have shown (Figure 7) that some new TE loci were shared between isolates and that in each case, the concerned isolates belonged to a same monophyletic cluster (Figure 4). The most parsimonious scenario is that TE insertions occurred in *M*. *incognita*, after the separation of the different main clusters but before the diversification of the phylogenetically related isolates, within a cluster, in a common ancestor. Other new TE loci, in contrast, were so far isolate‐specific, suggesting some TE insertions were even more recent, and that TE mobility might be a continuous phenomenon. No information is available about the ancientness of cultivated lands in Brazil on which the different isolates have been sampled. However, because there is no significant correlation between the isolates geographical distribution and the phylogenetic clusters, whether it is TE‐based (this study) or SNV‐based (Koutsovoulos, Marques et al., 2020), we can hypothesize these isolates have been recently spread by human agricultural activity in the last centuries.

Overall, the presence of lineage and isolate‐specific TE insertions, the distribution of percent identities of some TE copies to their consensuses shifted toward high value, and transcriptional support for some of the genes involved in the transposition machinery suggest TEs have recently been active in *M*. *incognita* and are possibly still active.

### 4.2 Functional impact of TE activity in *Meloidogyne incognita* and other nematodes

*Meloidogyne* *incognita* is a parthenogenetic mitotic nematode of major agronomic importance. This pest shows no sign of sexual recombination and only a few genome variations at the SNP level (Koutsovoulos, Marques et al., 2020). The molecular mechanisms underlying the genome plasticity necessary for adaptive evolution remain poorly known. In this study, we investigated whether TE movements could contribute to the *M*. *incognita* genome plasticity.

In *M*. *javanica*, a closely related root‐knot nematode, comparison between an avirulent line unable to infect tomato plants carrying a nematode resistance gene and another virulent line that overcame this resistance led to the identification of a gene present in the avirulent nematodes but absent from the virulent ones. Interestingly, the gene under consideration is present in a TIR‐like DNA transposon and its absence in the virulent line suggests this is due to excision of the transposon and thus that TE activity plays a role in *M*. *javanica* adaptive evolution (Gross & Williamson, 2011).

In *M*. *incognita*, convergent gene losses at the whole‐genome level between two virulent populations compared with their avirulent populations of origin were recently reported (Castagnone‐Sereno et al., 2019). Gene copy‐number variations (CNVs) are genome plasticity factors known to be involved in adaptive evolution (Katju & Bergthorsson, 2013), and TE can actively (e.g., by gene hitchhiking) or passively (e.g., through recombination) participate in these variations. This CNV analysis in *M*. *incognita* was done on a previous version of the genome (Abad et al., 2008), which was partially incomplete, and the possible contribution of TEs in these CNVs could not be assessed. Although the current version of the genome (Blanc‐Mathieu et al., 2017) is more complete and consistent with the estimated genome size, it is still fragmentary with thousands of scaffolds and a relatively low N50 length (38.6 kb). This fragmentation prevents a thorough identification of TE‐rich and TE‐poor regions and possible colocalization with CNV loci at the whole‐genome scale. Availability of long read‐based more contiguous genome assembly in the future will certainly allow reinvestigating CNV and the possible involvement of TEs in association with an adaptive process such as resistance breaking down.

As previously evoked, in *M*. *incognita* we found that the genome‐wide pattern of variations of TE frequencies across the loci between the different isolates recapitulated almost exactly their phylogeny built on SNV in coding regions (Figure 4). Hence, most of the divergence in terms of TE landscapes follows the divergence at the nucleotide level. Almost the same conclusion was drawn by comparing SNV and TE variation data across different *C*. *elegans* populations (Laricchia et al., 2017). In *M*. *incognita*, the phylogeny of isolates does not significantly correlate with the monitored biological traits, namely geographical distribution, range of compatible host plants, and nature of the crop currently infected (Koutsovoulos, Marques et al., 2020). Interestingly, no correlation was also observed between variations in TE frequencies and geographical distribution for European *Drosophila* populations (Lerat et al., 2019). The lack of evident correlation between the phylogenetic signal, regardless of whether it is TE‐based or SNV‐based, and the biological traits under consideration suggests most of the variations follow the drift between isolates and are not necessarily adaptive, which is not surprising. A similar conclusion was also drawn recently by analyzing 625 fungal genomes and observing that most TE movements were presumably neutral and adaptive ones being marginal (Muszewska et al., 2019).

On another note, as explained in the first section of the discussion, TE activity is possibly very recent in *M*. *incognita* and this might contribute to the current lack of evidence for association between TE activity, including invasion or decay across isolates, and adaptive traits.

Yet, we detected and confirmed by PCR insertions of some TEs inside genes or possible regulatory regions. We found that more than 90% of the TEs involved were TIRs or MITEs, which echoes their enrichment among the most active TEs in *M*. *incognita*. In the Mulberry genome, MITEs inserted near genes were shown to regulate gene expression via small RNAs, while those inserted within genes were associated with alternative splice variants (Xin et al., 2019). Similarly, in the wheat genome, MITEs of the mariner superfamily played an instrumental role in generating the diversity of micro‐RNAs involved in important adaptive traits such as resistance to pathogens (Poretti et al., 2020). The exact functional impact of TE insertions in *M*. *incognita* would need to be evaluated in the future. Generating transcriptomics data for the different isolates would enable studying associated differences in gene expression patterns or transcript diversity. As a complementary approach, proteomic studies would allow direct search for differences at the encoded protein level.

In *M*. *incognita*, almost 23% of the genes have been described as specific to plant–parasite species, without any recognizable homology in other species (Grynberg et al., 2020). Interestingly, TE movements can be involved in the emergence of species or genus‐specific “orphan” genes (Jin et al., 2019; Ruiz‐Orera et al., 2015; Wu & Knudson, 2018). Because some of the *M*. *incognita* genes impacted by TE insertions are specific to plant–parasite species and yet widely conserved among these parasites, a role in plant parasitism is possible.

### 4.3 Ploidy, (a)sexuality, and hybridization: a complex interplay influencing TE load and composition

*Meloidogyne* *incognita* is an asexual (mitotic parthenogenetic), polyploid, and hybrid species. These three features are expected to impact TE load in the genome with various intensities and possibly conflicting effects.

Contradictory theories exist concerning the activity/proliferation of TEs as a function of the reproductive mode. The higher efficacy of selection under sexual reproduction can be viewed as an efficient system to purge TEs and control their proliferation. Supporting these views, in parasitoid wasps, TE load was shown to be higher in asexual lineages, induced by the endosymbiotic *Wolbachia* bacteria, than in sexual lineages (Kraaijeveld et al., 2012). However, whether this higher load is a consequence of the shift in reproductive mode or of *Wolbachia* infection remains to be clarified.

In an opposite theory, sexual reproduction can also be considered as a way for TEs to spread across individuals within the population, whereas in clonal reproduction, the transposons are trapped exclusively in the offspring of the holding individual. Under this view, asexual reproduction is predicted to reduce TE load as TEs are unable to spread in other individuals, and are thus removed by genetic drift and/or purifying selection in the long term (Wright & Finnegan, 2001). Consistent with this theory, comparison of sexual and asexual *Saccharomyces cerevisiae* populations showed that the TE load decreases rapidly under asexual reproduction (Bast et al., 2019).

Hence, whether the TE load is expected to be higher or lower in clonal species compared with sexual relatives remains unclear and other conflicting factors such as TE excision rate and the effective size of the population probably blur the signal (Glémin et al., 2019). The breeding system has been shown to constitute an important factor influencing TE distribution in *Caenorhabditis* genomes (Dolgin et al., 2008): TEs in self‐fertilizing populations seem to be selectively neutral and segregate at higher frequency than in outcrossing populations, where they are submitted to purifying selection. Interestingly, at a broader scale, a comparative analysis of different lineages of sexual and asexual arthropods revealed no evidence for differences in TE load according to the reproductive modes (Bast et al., 2015). Similar conclusions were drawn at the whole nematoda phylum scale (Szitenberg et al., 2016), although only one apomictic asexually reproducing species (i.e., *M*. *incognita*) was present in the comparative analysis.

Polyploidy, in contrast, is commonly accepted as a major event initially favouring the multiplication and activity of TEs. This is clearly described with numerous examples in plants (Vicient & Casacuberta, 2017), and some examples are also emerging in animals (Rodriguez & Arkhipova, 2018). When hybridization and polyploidy are combined, this can lead to TE bursts in the genome. As originally proposed by Barbara McClintock, allopolyploidization produces a “genomic shock,” a genome instability associated with the relaxation of the TE silencing mechanisms and the reactivation of ancient TEs (McClintock, 1984; Mhiri et al., 2019).

Hybridization, polyploidy, and asexual reproduction are combined in *M*. *incognita* with relative effects on the TE load extremely challenging, if not impossible, to disentangle. Comparisons of the TE loads in three allopolyploid clonal *Meloidogyne* (*M*. *incognita*, *M*. *javanica*, and *M*. *arenaria*) against a diploid facultative sexual relative (*M*. *hapla*) suggested a higher TE load in the clonal species (Blanc‐Mathieu et al., 2017). However, the TE annotation and filtering strategy used was different from our current work and not directly comparable. More recently, the genome of *M*. *graminicola*, another meiotic diploid and facultative sexual species, was annotated for TEs using the exact same strategy than the one used here for *M*. *incognita* (Phan et al., 2020). The study showed canonical TE occupied a lower proportion of the *M*. *graminicola* genome (~2.6%) than in *M*. *incognita* (~4.7%). Nonetheless, to differentiate the relative contribution of polyploidy, hybridization, and reproductive mode to the *M*. *incognita* TE load, it would be necessary to conduct comparative analysis with a same method, on polyploid sexuals and on diploid asexuals in the genus *Meloidogyne*, and ideally with and without hybrid origin. So far, additional genomic sequences are only available for other polyploid clonal species, which are all suspected to have a hybrid origin (Blanc‐Mathieu et al., 2017; Koutsovoulos, Poullet et al., 2020; Susič et al., 2020; Szitenberg et al., 2017). Hence, further sampling of *Meloidogyne* species with diverse ploidy levels and reproductive modes will be necessary to disentangle the relative contribution of these three features on the TE abundance and composition.

### 4.4 Concluding remarks

In this study, we used population genomics technique and statistical analyses of the results to assess whether TE could contribute to the genome dynamics of *M*. *incognita* and possibly to its adaptive evolution. Overall, we provided a body of evidence suggesting TEs have been at least recently active and might still be active. With thousands of loci showing variations in TE presence frequencies across geographical isolates, there is a clear impact on the *M*. *incognita* genome plasticity. Being inserted in coding or regulatory regions, some TE might have a functional impact. Most of the genes in these species are functionally uncharacterized so far and those impacted by TE insertions will deserve further analyses to assess the functional impact of TE movements. This pioneering study constitutes a valuable resource and opens new perspectives for future targeted investigation of the potential effect of TE dynamics on the evolution, fitness, and adaptability of *M*. *incognita* and in the whole nematoda phylum.

## CONFLICT OF INTEREST

The authors of this paper declare that they have no financial conflict of interest with the content of this article.

## Supporting information

Supplementary Material

## Data Availability

All the raw and filtered data generated in this study, as well as details of the experimental procedures, scripts, and datasets, have been deposited and made publicly available in the institutional INRAE Data Portal at this URL: https://data.inrae.fr/dataverse/TE‐mobility‐in‐MiV3 and cited throughout the text where appropriate, with DOIs available in the references.

## References

[eva13246-bib-0001] Abad, P. , Gouzy, J. , Aury, J.‐M. , Castagnone‐Sereno, P. , Danchin, E. G. J. , Deleury, E. , Perfus‐Barbeoch, L. , Anthouard, V. , Artiguenave, F. , Blok, V. C. , Caillaud, M.‐C. , Coutinho, P. M. , Dasilva, C. , De Luca, F. , Deau, F. , Esquibet, M. , Flutre, T. , Goldstone, J. V. , Hamamouch, N. , … Wincker, P. (2008). Genome sequence of the metazoan plant‐parasitic nematode Meloidogyne incognita. Nature Biotechnology, 26, 909–915. 10.1038/nbt.1482 18660804

[eva13246-bib-0002] Agrios, G. N. (2005). Plant pathology (5th ed.). Elsevier Academic Press.

[eva13246-bib-0003] Aminetzach, Y. T. , Macpherson, J. M. , & Petrov, D. A. (2005). Pesticide resistance via transposition‐mediated adaptive gene truncation in Drosophila. Science, 309, 764–767. 16051794 10.1126/science.1112699

[eva13246-bib-0004] Andrews, S. (2010). FastQC: A quality control tool for high throughput sequence data [Online]. http://www.bioinformatics.babraham.ac.uk/projects/fastqc/

[eva13246-bib-0005] Ansaloni, F. , Scarpato, M. , Di Schiavi, E. , Gustincich, S. , & Sanges, R. (2019). Exploratory analysis of transposable elements expression in the *C. elegans* early embryo. BMC Bioinformatics, 20, 484. 31757208 10.1186/s12859-019-3088-7 PMC6873666

[eva13246-bib-0006] Anxolabéhère, D. , Kidwell, M. G. , & Periquet, G. (1988). Molecular characteristics of diverse populations are consistent with the hypothesis of a recent invasion of *Drosophila melanogaster* by mobile P elements. Molecular Biology and Evolution, 5, 252–269. 2838720 10.1093/oxfordjournals.molbev.a040491

[eva13246-bib-0007] Barbary, A. , Djian‐Caporalino, C. , Palloix, A. , & Castagnone‐Sereno, P. (2015). Host genetic resistance to root‐knot nematodes, Meloidogyne spp., in Solanaceae: From genes to the field. Pest Management Science, 71, 1591–1598. 26248710 10.1002/ps.4091

[eva13246-bib-0008] Bast, J. , Jaron, K. S. , Schuseil, D. , Roze, D. , & Schwander, T. (2019). Asexual reproduction reduces transposable element load in experimental yeast populations. Coop G, Tautz D, Coop G, Charlesworth B, editors. eLife, 8, e48548. 31486772 10.7554/eLife.48548 PMC6783261

[eva13246-bib-0009] Bast, J. , Schaefer, I. , Schwander, T. , Maraun, M. , Scheu, S. , & Kraaijeveld, K. (2015). No accumulation of transposable elements in asexual arthropods. Molecular Biology and Evolution, 33(3), msv261. 10.1093/molbev/msv261 PMC4760076 26560353

[eva13246-bib-0010] Bégin, M. , & Schoen, D. J. (2007). Transposable elements, mutational correlations, and population divergence in *Caenorhabditis elegans* . Evolution, 61, 1062–1070. 17492961 10.1111/j.1558-5646.2007.00097.x

[eva13246-bib-0011] Belyayev, A. (2014). Bursts of transposable elements as an evolutionary driving force. Journal of Evolutionary Biology, 27, 2573–2584. 25290698 10.1111/jeb.12513

[eva13246-bib-0012] Bessereau, J.‐L. (2006). Transposons in *C. elegans* . *WormBook* [Internet]. Available from: http://www.wormbook.org/chapters/www_transposons/transposons.html 10.1895/wormbook.1.70.1 PMC4781069 18023126

[eva13246-bib-0013] Blanc‐Mathieu, R. , Perfus‐Barbeoch, L. , Aury, J.‐M. , Da Rocha, M. , Gouzy, J. , Sallet, E. , Martin‐Jimenez, C. , Bailly‐Bechet, M. , Castagnone‐Sereno, P. , Flot, J.‐F. , Kozlowski, D. K. , Cazareth, J. , Couloux, A. , Da Silva, C. , Guy, J. , Kim‐Jo, Y.‐J. , Rancurel, C. , Schiex, T. , Abad, P. , … Danchin, E. G. J. (2017). Hybridization and polyploidy enable genomic plasticity without sex in the most devastating plant‐parasitic nematodes. PLoS Genetics, 13, e1006777. 28594822 10.1371/journal.pgen.1006777 PMC5465968

[eva13246-bib-0014] Bourgeois, Y. , & Boissinot, S. (2019). On the population dynamics of junk: A review on the population genomics of transposable elements. Genes, 10, 419. 10.3390/genes10060419 PMC6627506 31151307

[eva13246-bib-0015] Castagnone‐Sereno, P. (2006). Genetic variability and adaptive evolution in parthenogenetic root‐knot nematodes. Heredity, 96, 282–289. 16404412 10.1038/sj.hdy.6800794

[eva13246-bib-0016] Castagnone‐Sereno, P. , & Danchin, E. G. J. (2014). Parasitic success without sex – The nematode experience. Journal of Evolutionary Biology, 27, 1323–1333. 25105196 10.1111/jeb.12337

[eva13246-bib-0017] Castagnone‐Sereno, P. , Mulet, K. , Danchin, E. G. J. , Koutsovoulos, G. D. , Karaulic, M. , Rocha, M. D. , Bailly‐Bechet, M. , Pratx, L. , Perfus‐Barbeoch, L. , & Abad, P. (2019). Gene copy number variations as signatures of adaptive evolution in the parthenogenetic, plant‐parasitic nematode *Meloidogyne incognita* . Molecular Ecology, 28, 2559–2572. 30964953 10.1111/mec.15095

[eva13246-bib-0018] Castagnone‐Sereno, P. , Wajnberg, E. , Bongiovanni, M. , Leroy, F. , & Dalmasso, A. (1994). Genetic variation in *Meloidogyne incognita* virulence against the tomatoMi resistance gene: evidence from isofemale line selection studies. TAG. Theoretical and Applied Genetics, 88, 749–753. 24186172 10.1007/BF01253980

[eva13246-bib-0019] Danchin, E. , & Da Rocha, M. (2020). *M. incognita* protein‐coding genes expression patterns. *Portail Data INRAE* [Internet]. Available from: 10.15454/YM2DHE

[eva13246-bib-0020] Dobin, A. , Davis, C. A. , Schlesinger, F. , Drenkow, J. , Zaleski, C. , Jha, S. , Batut, P. , Chaisson, M. , & Gingeras, T. R. (2013). STAR: Ultrafast universal RNA‐seq aligner. Bioinformatics, 29, 15–21. 23104886 10.1093/bioinformatics/bts635 PMC3530905

[eva13246-bib-0021] Dolgin, E. S. , Charlesworth, B. , & Cutter, A. D. (2008). Population frequencies of transposable elements in selfing and outcrossing Caenorhabditis nematodes. Genetical Research, 90, 317–329. 10.1017/S0016672308009440 18840306

[eva13246-bib-0022] Emmons, S. W. , & Yesner, L. (1984). High‐frequency excision of transposable element Tc1 in the nematode *Caenorhabditis elegans* is limited to somatic cells. Cell, 36, 599–605. 6321037 10.1016/0092-8674(84)90339-8

[eva13246-bib-0023] Faino, L. , Seidl, M. F. , Shi‐Kunne, X. , Pauper, M. , van den Berg, G. C. M. , Wittenberg, A. H. J. , & Thomma, B. P. H. J. (2016). Transposons passively and actively contribute to evolution of the two‐speed genome of a fungal pathogen. Genome Research, 26, 1091–1100. 27325116 10.1101/gr.204974.116 PMC4971763

[eva13246-bib-0024] Finn, R. D. , Coggill, P. , Eberhardt, R. Y. , Eddy, S. R. , Mistry, J. , Mitchell, A. L. , Potter, S. C. , Punta, M. , Qureshi, M. , Sangrador‐Vegas, A. , Salazar, G. A. , Tate, J. , & Bateman, A. (2016). The Pfam protein families database: Towards a more sustainable future. Nucleic Acids Research, 44, D279–D285. 26673716 10.1093/nar/gkv1344 PMC4702930

[eva13246-bib-0025] Flutre, T. , Duprat, E. , Feuillet, C. , & Quesneville, H. (2011). Considering transposable element diversification in de novo annotation approaches. PLoS One, 6, e16526. 21304975 10.1371/journal.pone.0016526 PMC3031573

[eva13246-bib-0026] Glémin, S. , François, C. M. , & Galtier, N. (2019). Genome evolution in outcrossing vs. selfing vs. asexual species. In M. Anisimova (Ed.), Evolutionary genomics: Statistical and computational methods. Methods in molecular biology (pp. 331–369). Springer. 10.1007/978-1-4939-9074-0_11 31278670

[eva13246-bib-0027] Gross, S. M. , & Williamson, V. M. (2011). Tm1: A mutator/foldback transposable element family in root‐knot nematodes. PLoS One, 6, e24534. 21931741 10.1371/journal.pone.0024534 PMC3169594

[eva13246-bib-0028] Grynberg, P. , Coiti Togawa, R. , Dias de Freitas, L. , Antonino, J. D. , Rancurel, C. , Mota do Carmo Costa, M. , Grossi‐de‐Sa, M. F. , Miller, R. N. G. , Brasileiro, A. C. M. , Messenberg Guimaraes, P. , & Danchin, E. G. J. (2020). Comparative genomics reveals novel target genes towards specific control of plant‐parasitic nematodes. Genes, 11, 1347. 10.3390/genes11111347 PMC7696266 33202889

[eva13246-bib-0029] Guerreiro, M. P. G. (2014). Interspecific hybridization as a genomic stressor inducing mobilization of transposable elements in *Drosophila* . Mobile Genetic Elements, 4, e34394. 25136509 10.4161/mge.34394 PMC4132227

[eva13246-bib-0030] Herrero, J. , Muffato, M. , Beal, K. , Fitzgerald, S. , Gordon, L. , Pignatelli, M. , Vilella, A. J. , Searle, S. M. J. , Amode, R. , Brent, S. , Spooner, W. , Kulesha, E. , Yates, A. , & Flicek, P. (2016). Ensembl comparative genomics resources. Database, 2016, bav096. 10.1093/database/bav096 26896847 PMC4761110

[eva13246-bib-0031] Hill, W. G. , & Robertson, A. (1966). The effect of linkage on limits to artificial selection. Genetical Research, 8, 269–294. 5980116

[eva13246-bib-0032] Hoffmann, A. A. , Reynolds, K. T. , Nash, M. A. , & Weeks, A. R. (2008). A high incidence of parthenogenesis in agricultural pests. Proceedings of the Royal Society of London. Series B: Biological Sciences, 275, 2473–2481. 18647717 10.1098/rspb.2008.0685 PMC2603198

[eva13246-bib-0033] Jin, G.‐H. , Zhou, Y.‐L. , Yang, H. , Hu, Y.‐T. , Shi, Y. , Li, L. , Siddique, A. N. , Liu, C.‐N. , Zhu, A.‐D. , Zhang, C.‐J. , & Li, D.‐Z. (2019). Genetic innovations: Transposable element recruitment and de novo formation lead to the birth of orphan genes in the rice genome. Journal of Systematics and Evolution, 59(2), 341–351. https://onlinelibrary.wiley.com/doi/abs/10.1111/jse.12548

[eva13246-bib-0034] Jones, J. T. , Haegeman, A. , Danchin, E. G. J. , Gaur, H. S. , Helder, J. , Jones, M. G. K. , Kikuchi, T. , Manzanilla‐López, R. , Palomares‐Rius, J. E. , Wesemael, W. M. L. , & Perry, R. N. (2013). Top 10 plant‐parasitic nematodes in molecular plant pathology. Molecular Plant Pathology, 14, 946–961. 23809086 10.1111/mpp.12057 PMC6638764

[eva13246-bib-0035] Kanazawa, A. , Liu, B. , Kong, F. , Arase, S. , & Abe, J. (2009). Adaptive evolution involving gene duplication and insertion of a novel Ty1/copia‐like retrotransposon in soybean. Journal of Molecular Evolution, 69, 164–175. 19629571 10.1007/s00239-009-9262-1

[eva13246-bib-0036] Katju, V. , & Bergthorsson, U. (2013). Copy‐number changes in evolution: Rates, fitness effects and adaptive significance. Frontiers in Genetics [Internet], 4, 273. Available from: http://www.frontiersin.org/Evolutionary_and_Population_Genetics/10.3389/fgene.2013.00273/abstract 24368910 10.3389/fgene.2013.00273 PMC3857721

[eva13246-bib-0037] Kazazian, H. H. , Wong, C. , Youssoufian, H. , Scott, A. F. , Phillips, D. G. , & Antonarakis, S. E. (1988). Haemophilia A resulting from de novo insertion of L 1 sequences represents a novel mechanism for mutation in man. Nature, 332, 164–166. 2831458 10.1038/332164a0

[eva13246-bib-0038] Kofler, R. , Gómez‐Sánchez, D. , & Schlötterer, C. (2016). PoPoolationTE2: Comparative population genomics of transposable elements using Pool‐Seq. Molecular Biology and Evolution, 33, 2759–2764. 27486221 10.1093/molbev/msw137 PMC5026257

[eva13246-bib-0039] Kondrashov, A. S. (1988). Deleterious mutations and the evolution of sexual reproduction. Nature, 336, 435–440. 3057385 10.1038/336435a0

[eva13246-bib-0040] Koutsovoulos, G. D. , Marques, E. , Arguel, M.‐J. , Duret, L. , Machado, A. C. Z. , Carneiro, R. M. D. G. , Kozlowski, D. K. , Bailly‐Bechet, M. , Castagnone‐Sereno, P. , Albuquerque, E. V. S. , & Danchin, E. G. J. (2020). Population genomics supports clonal reproduction and multiple independent gains and losses of parasitic abilities in the most devastating nematode pest. Evolutionary Applications, 13, 442–457. 31993088 10.1111/eva.12881 PMC6976969

[eva13246-bib-0041] Koutsovoulos, G. D. , Poullet, M. , Elashry, A. , Kozlowski, D. , Sallet, E. , Da Rocha, M. , Perfus‐Barbeoch, L. , Martin‐Jimenez, C. , Frey, J. E. , Ahrens, C. H. , Kiewnick, S. , & Danchin, E. (2020). Genome assembly and annotation of *Meloidogyne enterolobii*, an emerging parthenogenetic root‐knot nematode. Scientific Data, 7, 324. 10.1038/s41597-020-00666-0 33020495 PMC7536185

[eva13246-bib-0043] Kozlov, A. M. , Darriba, D. , Flouri, T. , Morel, B. , & Stamatakis, A. (2019). RAxML‐NG: A fast, scalable and user‐friendly tool for maximum likelihood phylogenetic inference. Bioinformatics, 35, 4453–4455. 31070718 10.1093/bioinformatics/btz305 PMC6821337

[eva13246-bib-0044] Kozlowski, D. (2020a). Transposable Elements prediction and annotation in the *M. incognita* genome. *Portail Data INRAE* [Internet]. Available from: 10.15454/EPTDOS

[eva13246-bib-0045] Kozlowski, D. (2020b). Transposable Elements prediction and annotation in the *C. elegans* genome. *Portail Data INRAE* [Internet]. Available from: 10.15454/LQCIW0

[eva13246-bib-0046] Kozlowski, D. (2020c). Contribution des éléments transposables à l’adaptabilité de ravageurs de cultures en absence de reproduction sexuée. Chapter VII, Available from: https://tel.archives‐ouvertes.fr/tel‐03153715

[eva13246-bib-0047] Kozlowski, D. (2020d). TE polymorphisms detection and analysis with PopoolationTE2. *Portail Data INRAE* [Internet]. Available from: 10.15454/EWJCT8

[eva13246-bib-0048] Kozlowski, D. , Da Rocha, M. , & Danchin, E. (2020). TE‐related genes: Annotation, characterisation, and expression. *Portail Data INRAE* [Internet]. Available from: 10.15454/DLDJVF

[eva13246-bib-0049] Kozlowski, D. , Hassanaly‐Goulamhoussen, R. , & Danchin, E. (2020). Experimental validations of TE‐impacted coding or regulatory loci. *Portail Data INRAE* [Internet]. Available from: 10.15454/NQAF31

[eva13246-bib-0050] Kraaijeveld, K. , Zwanenburg, B. , Hubert, B. , Vieira, C. , De Pater, S. , Van Alphen, J. J. M. , Den Dunnen, J. T. , & De Knijff, P. (2012). Transposon proliferation in an asexual parasitoid. Molecular Ecology, 21, 3898–3906. 22548357 10.1111/j.1365-294X.2012.5582.x

[eva13246-bib-0051] Kumar, S. , Stecher, G. , Suleski, M. , & Hedges, S. B. (2017). TimeTree: A resource for timelines, timetrees, and divergence times. Molecular Biology and Evolution, 34, 1812–1819. 28387841 10.1093/molbev/msx116

[eva13246-bib-0052] Laricchia, K. M. , Zdraljevic, S. , Cook, D. E. , & Andersen, E. C. (2017). Natural variation in the distribution and abundance of transposable elements across the *Caenorhabditis elegans* species. Molecular Biology and Evolution, 34, 2187–2202. 28486636 10.1093/molbev/msx155 PMC5850821

[eva13246-bib-0053] Lerat, E. , Goubert, C. , Guirao‐Rico, S. , Merenciano, M. , Dufour, A.‐B. , Vieira, C. , & González, J. (2019). Population‐specific dynamics and selection patterns of transposable element insertions in European natural populations. Molecular Ecology, 28, 1506–1522. 30506554 10.1111/mec.14963 PMC6849870

[eva13246-bib-0054] Letunic, I. , & Bork, P. (2019). Interactive tree of life (iTOL) v4: Recent updates and new developments. Nucleic Acids Research, 47, W256–W259. 30931475 10.1093/nar/gkz239 PMC6602468

[eva13246-bib-0055] Li, B. , & Dewey, C. N. (2011). RSEM: Accurate transcript quantification from RNA‐Seq data with or without a reference genome. BMC Bioinformatics, 12, 323. 21816040 10.1186/1471-2105-12-323 PMC3163565

[eva13246-bib-0056] Li, H. , & Durbin, R. (2009). Fast and accurate short read alignment with Burrows‐Wheeler transform. Bioinformatics, 25, 1754–1760. 19451168 10.1093/bioinformatics/btp324 PMC2705234

[eva13246-bib-0057] Li, H. , Handsaker, B. , Wysoker, A. , Fennell, T. , Ruan, J. , Homer, N. , Marth, G. , Abecasis, G. , & Durbin, R. (2009). The sequence alignment/map format and SAMtools. Bioinformatics, 25, 2078–2079. 19505943 10.1093/bioinformatics/btp352 PMC2723002

[eva13246-bib-0058] Lively, C. M. (2010). A review of red queen models for the persistence of obligate sexual reproduction. Journal of Heredity, 101, S13–S20. 10.1093/jhered/esq010 20421322

[eva13246-bib-0059] Llorens, C. , Futami, R. , Covelli, L. , Dominguez‐Escriba, L. , Viu, J. M. , Tamarit, D. , Aguilar‐Rodriguez, J. , Vicente‐Ripolles, M. , Fuster, G. , Bernet, G. P. , Maumus, F. , Munoz‐Pomer, A. , Sempere, J. M. , Latorre, A. , & Moya, A. (2011). The Gypsy Database (GyDB) of mobile genetic elements: Release 2.0. Nucleic Acids Research, 39, D70–D74. 21036865 10.1093/nar/gkq1061 PMC3013669

[eva13246-bib-0060] Lu, L. , Chen, J. , Robb, S. M. C. , Okumoto, Y. , Stajich, J. E. , & Wessler, S. R. (2017). Tracking the genome‐wide outcomes of a transposable element burst over decades of amplification. Proceedings of the National Academy of Sciences, 114, E10550–E10559. 10.1073/pnas.1716459114 PMC5724284 29158416

[eva13246-bib-0061] Magwire, M. M. , Bayer, F. , Webster, C. L. , Cao, C. , & Jiggins, F. M. (2011). Successive increases in the resistance of drosophila to viral infection through a transposon insertion followed by a duplication. PLoS Genetics, 7, e1002337. 22028673 10.1371/journal.pgen.1002337 PMC3197678

[eva13246-bib-0062] Martin, M. (2011). Cutadapt removes adapter sequences from high‐throughput sequencing reads. EMBnet.journal, 17, 10–12.

[eva13246-bib-0063] McCarter, J. P. (2009). Molecular approaches toward resistance to plant‐parasitic nematodes. In R. H. Berg & C. G. Taylor (Eds.), Cell biology of plant nematode parasitism. Vol. 15. Plant cell monographs (pp. 239–267). Springer Berlin Heidelberg. Available from: http://www.springerlink.com/index/10.1007/978‐3‐540‐85215‐5_9

[eva13246-bib-0064] McClintock, B. (1984). The significance of responses of the genome to challenge. Science, 226, 792–801. 15739260 10.1126/science.15739260

[eva13246-bib-0065] Mhiri, C. , Parisod, C. , Daniel, J. , Petit, M. , Lim, K. Y. , de Borne, F. D. , Kovarik, A. , Leitch, A. R. , & Grandbastien, M.‐A. (2019). Parental transposable element loads influence their dynamics in young Nicotiana hybrids and allotetraploids. New Phytologist, 221, 1619–1633. 10.1111/nph.15484 30220091

[eva13246-bib-0066] Miki, Y. , Nishisho, I. , Horii, A. , Miyoshi, Y. , Utsunomiya, J. , Kinzler, K. W. , Vogelstein, B. , & Nakamura, Y. (1992). Disruption of the APC gene by a retrotransposal insertion of L1 sequence in a colon cancer. Cancer Research, 52, 643–645. 1310068

[eva13246-bib-0067] Muller, H. J. (1964). The relation of recombination to mutational advance. Mutation Research, 106, 2–9. 14195748 10.1016/0027-5107(64)90047-8

[eva13246-bib-0068] Muszewska, A. , Steczkiewicz, K. , Stepniewska‐Dziubinska, M. , & Ginalski, K. (2019). Transposable elements contribute to fungal genes and impact fungal lifestyle. Scientific Reports, 9, 4307. 30867521 10.1038/s41598-019-40965-0 PMC6416283

[eva13246-bib-0069] Paradis, E. , & Schliep, K. (2019). ape 5.0: An environment for modern phylogenetics and evolutionary analyses in R. Bioinformatics, 35, 526–528. 30016406 10.1093/bioinformatics/bty633

[eva13246-bib-0070] Pereira, V. , Enard, D. , & Eyre‐Walker, A. (2009). The effect of transposable element insertions on gene expression evolution in rodents. PLoS ONE, 4, e4321. 10.1371/journal.pone.0004321 19183808 PMC2629548

[eva13246-bib-0071] Phan, N. T. , Orjuela, J. , Danchin, E. G. J. , Klopp, C. , Perfus‐Barbeoch, L. , Kozlowski, D. K. , Koutsovoulos, G. D. , Lopez‐Roques, C. , Bouchez, O. , Zahm, M. , Besnard, G. , & Bellafiore, S. (2020). Genome structure and content of the rice root‐knot nematode (*Meloidogyne graminicola*). Ecology and Evolution, 10, 11006–11021. 10.1002/ece3.6680 33144944 PMC7593179

[eva13246-bib-0072] Poretti, M. , Praz, C. R. , Meile, L. , Kälin, C. , Schaefer, L. K. , Schläfli, M. , Widrig, V. , Sanchez‐Vallet, A. , Wicker, T. , & Bourras, S. (2020). Domestication of high‐copy transposons underlays the wheat small RNA response to an obligate pathogen. Molecular Biology and Evolution, 37, 839–848. 31730193 10.1093/molbev/msz272 PMC7038664

[eva13246-bib-0073] Quesneville, H. , Bergman, C. M. , Andrieu, O. , Autard, D. , Nouaud, D. , Ashburner, M. , & Anxolabehere, D. (2005). Combined evidence annotation of transposable elements in genome sequences. PLoS Computational Biology, 1, 166–175. 16110336 10.1371/journal.pcbi.0010022 PMC1185648

[eva13246-bib-0074] Quinlan, A. R. , & Hall, I. M. (2010). BEDTools: A flexible suite of utilities for comparing genomic features. Bioinformatics, 26, 841–842. 20110278 10.1093/bioinformatics/btq033 PMC2832824

[eva13246-bib-0075] Rice, W. R. (2002). Experimental tests of the adaptive significance of sexual recombination. Nature Reviews Genetics, 3, 241–251. 10.1038/nrg760 11967549

[eva13246-bib-0076] Rodriguez, F. , & Arkhipova, I. R. (2018). Transposable elements and polyploid evolution in animals. Current Opinion in Genetics & Development, 49, 115–123. 29715568 10.1016/j.gde.2018.04.003 PMC5975190

[eva13246-bib-0077] Ruiz‐Orera, J. , Hernandez‐Rodriguez, J. , Chiva, C. , Sabidó, E. , Kondova, I. , Bontrop, R. , Marqués‐Bonet, T. , & Albà, M. M. (2015). Origins of de novo genes in human and chimpanzee. PLoS Genetics, 11, e1005721. 26720152 10.1371/journal.pgen.1005721 PMC4697840

[eva13246-bib-0078] Savary, S. , Willocquet, L. , Pethybridge, S. J. , Esker, P. , McRoberts, N. , & Nelson, A. (2019). The global burden of pathogens and pests on major food crops. Nature Ecology & Evolution, 3, 430–439. 30718852 10.1038/s41559-018-0793-y

[eva13246-bib-0079] Stuart, T. , Eichten, S. R. , Cahn, J. , Karpievitch, Y. V. , Borevitz, J. O. , & Lister, R. (2016). Population scale mapping of transposable element diversity reveals links to gene regulation and epigenomic variation. eLife, 5, e20777. 27911260 10.7554/eLife.20777 PMC5167521

[eva13246-bib-0080] Susič, N. , Koutsovoulos, G. D. , Riccio, C. , Danchin, E. G. J. , Blaxter, M. L. , Lunt, D. H. , Strajnar, P. , Širca, S. , Urek, G. , & Stare, B. G. (2020). Genome sequence of the root‐knot nematode *Meloidogyne luci* . Journal of Nematology, 52, 1–5. 10.21307/jofnem-2020-025 PMC7266024 32180388

[eva13246-bib-0081] Szitenberg, A. , Cha, S. , Opperman, C. H. , Bird, D. M. , Blaxter, M. L. , & Lunt, D. H. (2016). Genetic drift, not life history or RNAi, determine long term evolution of transposable elements. Genome Biology and Evolution, 8, 2964–2978. 27566762 10.1093/gbe/evw208 PMC5635653

[eva13246-bib-0082] Szitenberg, A. , Salazar‐Jaramillo, L. , Blok, V. C. , Laetsch, D. R. , Joseph, S. , Williamson, V. M. , Blaxter, M. L. , & Lunt, D. H. (2017). Comparative genomics of apomictic root‐knot nematodes: Hybridization, ploidy, and dynamic genome change. Genome Biology and Evolution, 9, 2844–2861. 29036290 10.1093/gbe/evx201 PMC5737495

[eva13246-bib-0083] The C. elegans Genome Sequencing Consortium . (1998). Genome sequence of the nematode *C. elegans*: A platform for investigating biology. Science, 282, 2012–2018. 9851916 10.1126/science.282.5396.2012

[eva13246-bib-0084] Trudgill, D. L. , & Blok, V. C. (2001). Apomictic, polyphagous root‐knot nematodes: Exceptionally successful and damaging biotrophic root pathogens. Annual Review of Phytopathology, 39, 53–77. 10.1146/annurev.phyto.39.1.53 11701859

[eva13246-bib-0085] Untergasser, A. , Nijveen, H. , Rao, X. , Bisseling, T. , Geurts, R. , & Leunissen, J. A. M. (2007). Primer3Plus, an enhanced web interface to Primer3. Nucleic Acids Research, 35, W71–W74. 17485472 10.1093/nar/gkm306 PMC1933133

[eva13246-bib-0086] Vicient, C. M. , & Casacuberta, J. M. (2017). Impact of transposable elements on polyploid plant genomes. Annals of Botany, 120, 195–207. 28854566 10.1093/aob/mcx078 PMC5737689

[eva13246-bib-0087] Vrijenhoek, R. C. , & Parker, E. D. (2009). Geographical parthenogenesis: General purpose genotypes and frozen niche variation. In I. Schön , K. Martens , & P. Dijk (Eds.), Lost sex (pp. 99–131). Springer Netherlands. http://www.springerlink.com/index/10.1007/978‐90‐481‐2770‐2_6

[eva13246-bib-0088] Wicker, T. , Sabot, F. , Hua‐Van, A. , Bennetzen, J. L. , Capy, P. , Chalhoub, B. , Flavell, A. , Leroy, P. , Morgante, M. , Panaud, O. , Paux, E. , SanMiguel, P. , & Schulman, A. H. (2007). A unified classification system for eukaryotic transposable elements. Nature Reviews Genetics, 8, 973–982. 10.1038/nrg2165 17984973

[eva13246-bib-0089] Wright, S. , & Finnegan, D. (2001). Genome evolution: Sex and the transposable element. Current Biology, 11, R296–R299. 11369217 10.1016/s0960-9822(01)00168-3

[eva13246-bib-0090] Wu, B. , & Knudson, A. (2018). Tracing the de novo origin of protein‐coding genes in yeast. mBio [Internet], 9(4), e1024–18. https://mbio.asm.org/content/9/4/e01024‐18 10.1128/mBio.01024-18 PMC6069113 30065088

[eva13246-bib-0091] Xin, Y. , Ma, B. , Xiang, Z. , & He, N. (2019). Amplification of miniature inverted‐repeat transposable elements and the associated impact on gene regulation and alternative splicing in mulberry (*Morus notabilis*). Mobile DNA, 10, 27. 31289464 10.1186/s13100-019-0169-0 PMC6593561

[eva13246-bib-0092] Zeng, L. , Pederson, S. M. , Kortschak, R. D. , & Adelson, D. L. (2018). Transposable elements and gene expression during the evolution of amniotes. Mobile DNA, 9, 17. 10.1186/s13100-018-0124-5 29942365 PMC5998507

