# Supplementary material for: Movements of transposable elements contribute to the genomic plasticity and species diversification in an asexually reproducing nematode pest
Source: Evol Appl. 2021 May 15;14(7):1844–66. doi: 10.1111/eva.13246 (PMC8288018; doi:10.1111/eva.13246)
Supplement: Supplementary file 1 — Supplementary Material [file EVA-14-1844-s001.pdf]

Supplementary material:

Movements of transposable elements contribute to the genomic plasticity and species diversification in an asexually reproducing nematode pest

Djampa KL KOZLOWSKI, Rahim HASSANALY-GOULAMHOUSSEN, Martine DA ROCHA, Georgios KOUTSOVOULOS, Marc BAILLY-BECHET*, Etienne GJ DANCHIN*.

* co-last authors
Affiliation : Université Côte d'Azur, INRAE, CNRS, ISA, Sophia Antipolis, France

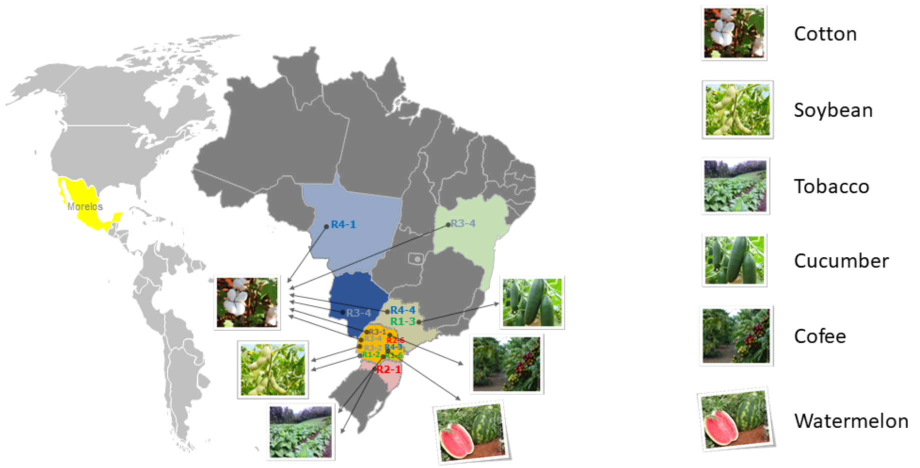

Adapted from Evolutionary Applications, Volume: 13, Issue: 2, Pages: 442-457, First published: 19 October 2019, DOI: (10.1111/eva.12881)

Fig S1: Isolates geographical distribution and host plants.

American continent map showing the geographical distribution for all isolates used in the study. Expanded map of Brazil shows the states where the 11 isolates sequenced in (Koutsovoulos et al. 2020) were collected. Each state is highlighted with a different colour. The crops from which the samples were isolated are illustrated by photographs, which are pointed by arrows coming from the name of the respective isolate.

Table S1: Reads libraries accession numbers & statistics

Lib. name	acess. nb. (SRA)	nb. reads (P-E)	read length (bp)	% GC
morelos	ERS1696677	76077411	2*150	28
R1-2	SRX4373671	76359269	2*150	29
R1-3	SRX4373672	75542522	2*150	28
R1-6	SRX4373673	75033425	2*150	28
R2-1	SRX4373674	75065658	2*150	29
R2-6	SRX4373675	75300726	2*150	29
R3-1	SRX4373676	74468408	2*150	30
R3-2	SRX4373677	74671928	2*150	28
R3-4	SRX4373678	74620706	2*150	28
R4-1	SRX4373679	75063890	2*150	28
R4-3	SRX4373680	75235737	2*150	29
R4-4	SRX4373681	74987959	2*150	28

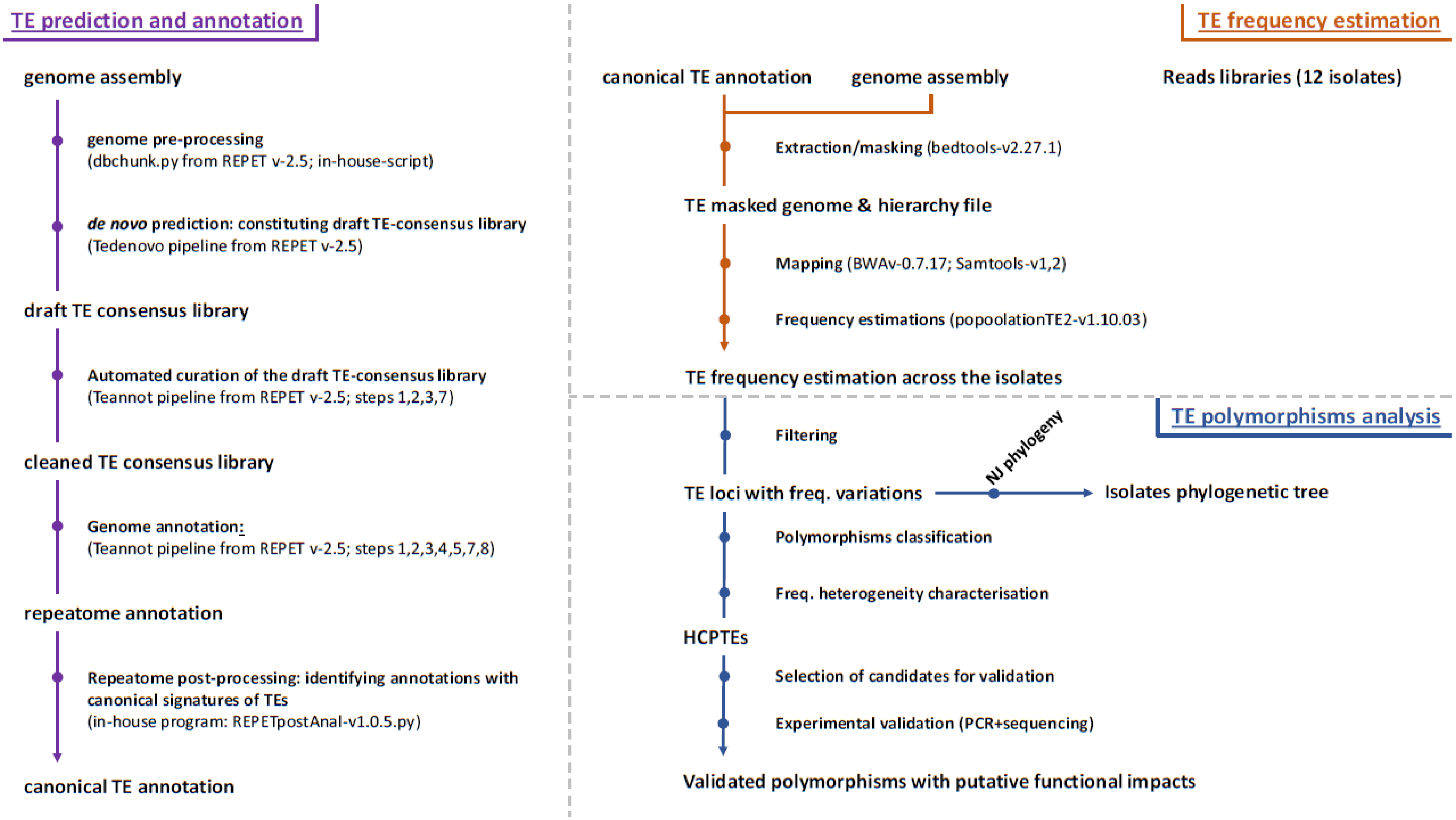
TE prediction and annotation
genome assembly
genome pre-processing
(dbchunk.py from REPET v-2.5; in-house-script)
de novo prediction: constituting draft TE-consensus library
(Tedenovo pipeline from REPET v-2.5)
draft TE consensus library
Automated curation of the draft TE-consensus library
(Teannot pipeline from REPET v-2.5; steps 1,2,3,7)
cleaned TE consensus library
Genome annotation:
(Teannot pipeline from REPET v-2.5; steps 1,2,3,4,5,7,8)
repeatome annotation
Repeatome post-processing: identifying annotations with canonical signatures of TEs
(in-house program: REPETpostAnal-v1.0.5.py)
canonical TE annotation
TE frequency estimation
canonical TE annotation
genome assembly
Reads libraries (12 isolates)
Extraction/masking (bedtools-v2.27.1)
TE masked genome & hierarchy file
Mapping (BWAv-0.7.17; Samtools-v1,2)
Frequency estimations (popoolationTE2-v1.10.03)
TE frequency estimation across the isolates
TE polymorphisms analysis
Filtering
TE loci with freq. variations
NJ phylogeny
Isolates phylogenetic tree
Polymorphisms classification
Freq. heterogeneity characterisation
HCPTEs
Selection of candidates for validation
Experimental validation (PCR+sequencing)
Validated polymorphisms with putative functional impacts

Fig S2: workflow overview.

The current analysis encompasses 3 pipelines: the TE prediction and annotation, the TE frequency estimation, and the TE polymorphisms analysis. Each step's workflow is represented in a separated panel. Each step of each sub-pipeline is explained in detail in Methods. All the scripts are available in (Kozlowski 2020). "Polymorphisms classification" and "Freq. heterogeneity characterisation" steps of the TE-polymorphism pipeline are detailed as a decision tree in Fig S8

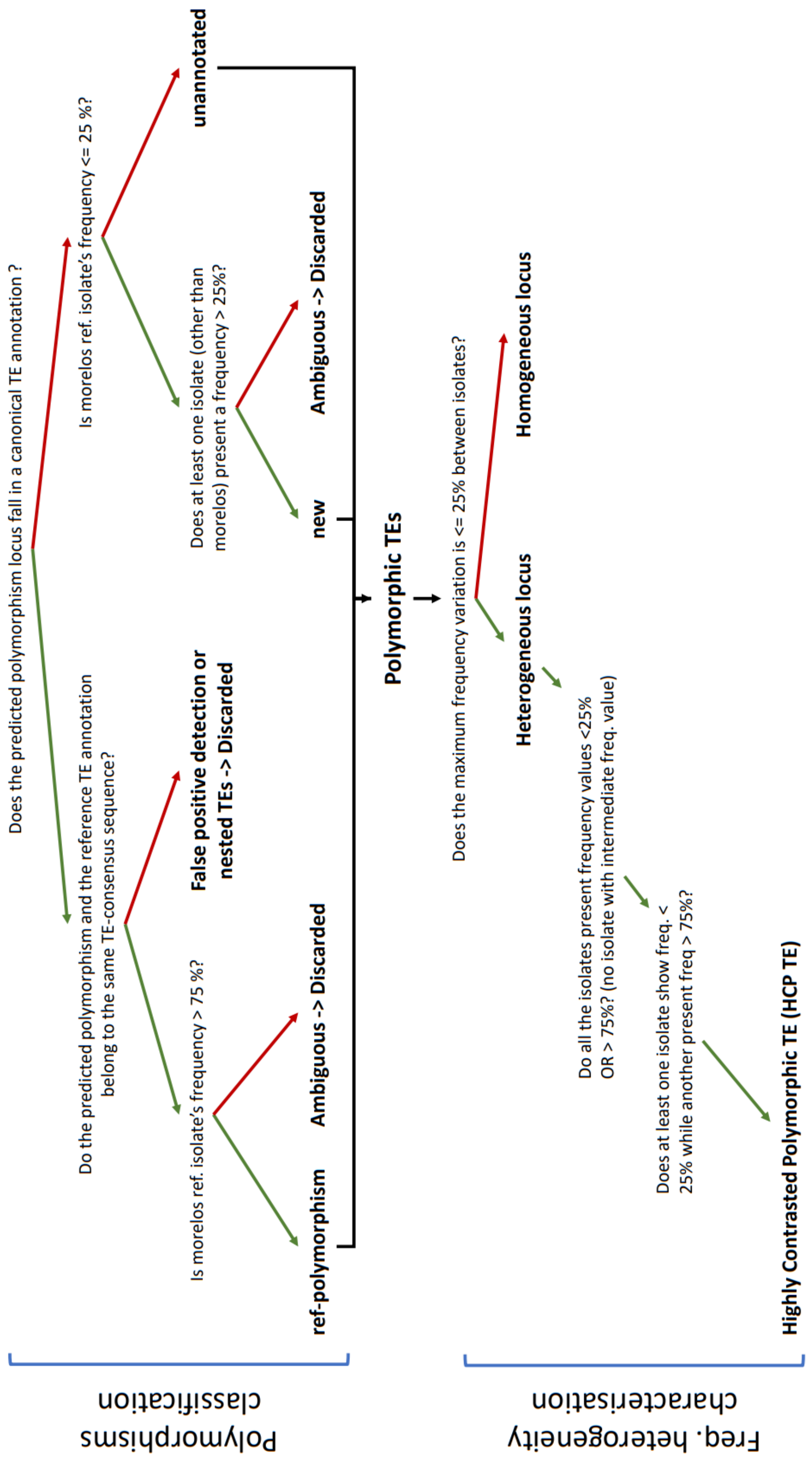

Fig S3: decision trees for polymorphisms classification and frequency heterogeneity characterisation.

This figure details as a decision tree the "Polymorphisms classification" and "Freq. heterogeneity characterisation" steps of the TE-polymorphism pipeline from the Fig S1. Green arrows represent a positive answer. The red ones represent a negative answer.

Table S2: PCR primers targeting 5 candidate locus for TE insertion

Primer Pair	Sequence	Amplicon size without insertion (bp)	Amplicon size with insertion (bp)
Locus1-F	CTTAGGTTTTTGACTGCGTCTGCCAT	180	973
Locus1-R	CAGATGCATTGCGGTGACGTTCTT		
Locus2-F	GGGGGTCAGATTACCCTCTATTATGGCA	761	1870
Locus2-R	CCTCTCCCATCACTCTCACAACCCA		
Locus3-F	CCGTCGGCGGGATCCCTGATATAAA	690	1814
Locus3-R	TTATCGGTTTCAACCCCGACCGAAC		
Locus4-F	GGTGGTGTTGTTGCTGGAATTACTAACC	981	1781
Locus4-R	GACAAACGTTGGAGCACGTTATGCTCG		
Locus5-F	GGAACAGTCAGCGGTGTCGGAAATC	1005	2080
Locus5-R	GTGTATGCTTCAGAACCCAGACGGGGA		
actin-F (ctrl +)	AAGATGGATGAAGAGGTAGCCGCCC	-	1667
actin-R (ctrl +)	ACTCTTGCTTGCTGATCCACCTTGA		

Table S3: Per-order summary of *M.incognita* draft TE annotations.

Autonomous TE orders (*) regroup elements known to present transposition machinery and thus able to transpose by themselves. On the opposite, non-autonomous orders (**) regroup elements lacking transposition machinery and therefore relying on autonomous elements to transpose. "Class 1 & 2 like" regroup elements for which homology-based evidence is sufficient to support an assignment to class I (retro) or II (DNA-transposon), but insufficient to assign a known order. "PotHostGenesOrOther" classification regroups elements which most likely correspond to duplicated genes. "Unclassif." elements are repetitive elements without sufficient evidence to be classified as class I (retro) or II (DNA-transposon). "Class 1 & 2 like", "PotHostGenesOrOther", and "Unclassif." are removed in the canonical TE annotations.

	order autonomous (*) / non-autonomous (**)	nb. of features	total length (bp)	genome percentage (%)	median length (bp)	median identity with consensus (%)
Retro - transposon	SINE (**)	19	6,618	0.004	258.0	87.6
	LARD (**)	217	132,969	0.072	244.0	92.35
	TRIM (**)	2,466	1,240,016	0.676	468.0	76.3
	LINE (*)	970	822,008	0.448	477.0	76.7
	LTR (*)	2,878	2,702,453	1.472	429.5	77.8
DNA - transposon	Helitron (*)	152	282,819	0.154	742.0	78.1
	Maverick (*)	17,684	9,553,119	5.205	364.0	74.8
	MITE (**)	12,435	5,126,098	2.793	363.0	88.5
	TIR (**)	11,094	5,389,275	2.936	379.0	85.0
Others	CLASS_1_LIKE	11,053	6,737,590	3.671	522.0	74.1
	CLASS_2_LIKE	77	34,339	0.019	497.0	98.7
	potHostGenesOrOther	26,225	12,185,975	6.640	359.0	75.1
	unclassif	8,811	4,212,017	2.295	390.0	79.0
	**Total**	**94,081**	**48,425,296**	**26.385**		

Table S4: Per-order summary of *M. incognita* ‘canonical’ TE annotations.

	order autonomous (*) / non-autonomous (**)	nb. of features	total length (bp)	genome percentage (%)	median length (bp)	median identity with consensus (%)
**Retro - transposon**	SINE (**)	9	4,522	0.002	528.0	99.7
	LARD (**)	45	6,342	0.035	1433.0	97.05
	TRIM (**)	174	104,018	0.057	525.0	97.7
	LINE (*)	145	313,224	0.171	1971.0	96.6
	LTR (*)	373	1,164,836	0.635	2415.0	97.0
**DNA - transposon**	Helitron (*)	18	86,666	0.047	5080.0	94.4
	Maverick (*)	189	1,307,068	0.712	6224.0	95.3
	MITE (**)	5085	2,755,381	1.501	525.0	96.2
	TIR (**)	3595	2,777,270	1.513	737.0	97.3
	**Total**	9,633	8,576,405	4.673		

Table S5: Per-order summary of *C.elegans* draft TE annotations.

	**order** autonomous (*) / non-autonomous (**)	**nb. of features**	**total length (bp)**	**genome percentage (%)**	**median length (bp)**	**median identity with consensus (%)**
**Retro - transposon**	SINE (**)	85	51,197	0.051	479.0	89.7
	LARD (**)	14	17,043	0.017	572.5	87.8
	TRIM (**)	3,324	2,184,226	2.178	485.0	79.1
	LINE (*)	519	480,089	0.479	538.0	96.4
	LTR (*)	246	215,384	0.215	509.0	96.15
**DNA - transposon**	Helitron (*)	2,865	2,103,981	2.098	547.0	77.2
	Maverick (*)	26	39,843	0.040	680.0	95.25
	MITE (**)	4,274	1,752,665	1.748	322.0	81.4
	TIR (**)	3,840	2,499,195	2.492	413.0	90.45
**Others**	CLASS_1_LIKE	46	19,873	0.020	385.5	85.325
	CLASS_2_LIKE	5,607	1,678,689	1.674	230.0	81.3
	potHostGenesOr Other	742	497,310	0.496	407.0	75.0
	unclassif	372	314,317	0.313	729.5	92.0
	**Total**	**21,960**	**11,853,812**	**11.820**		

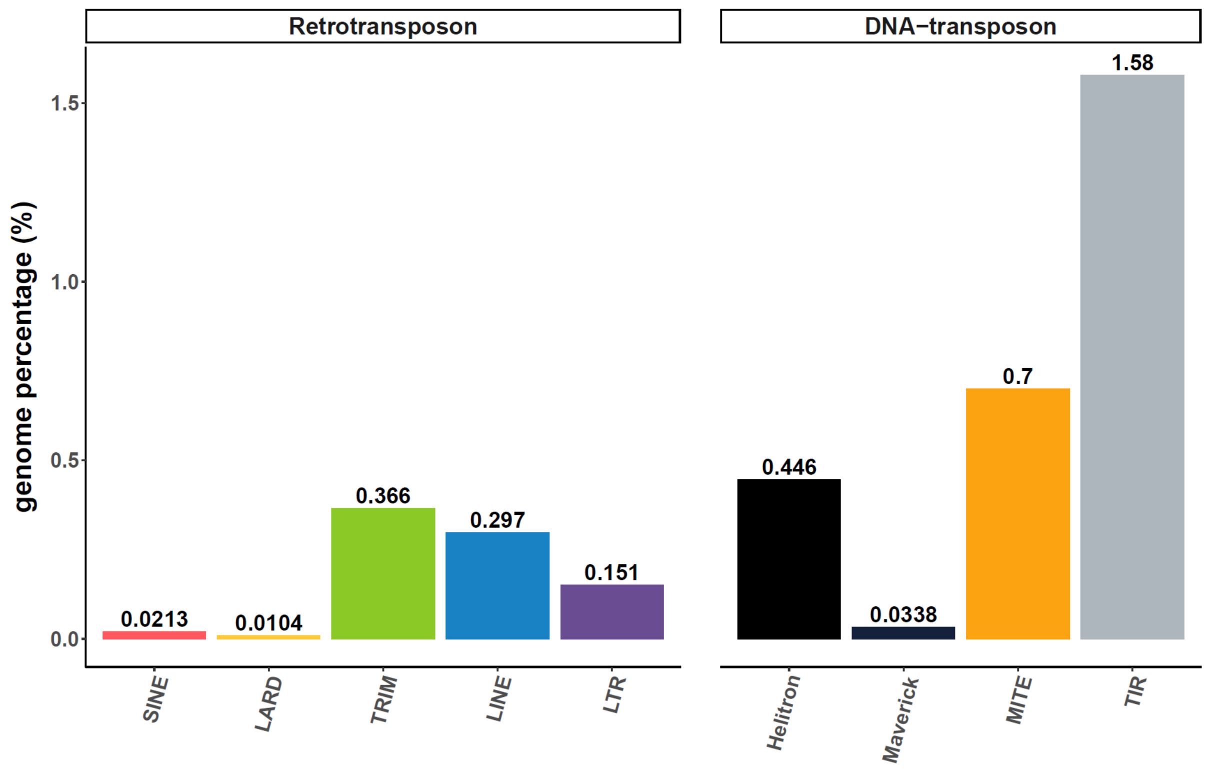

Fig S4: Canonical TE annotations distribution in the *C. elegans* genome

Genome percentage is based on a *C. elegans* genome size of 100,286,401 bp. More detailed statistics are available in supp. Table S6.

Table S6: Per-order summary of *C.elegans* canonical TE annotations.

	**order** autonomous (*) / non-autonomous (**)	**nb. of features**	**total length (bp)**	**genome percentage (%)**	**median length (bp)**	**median identity with consensus (%)**
**Retro - transposon**	SINE (**)	23	21,342	0.021	908.0	98.1
	LARD (**)	3	10,417	0.010	3969.0	99.7
	TRIM (**)	294	366,742	0.366	744.5	90.6
	LINE (*)	184	297,840	0.297	1252.5	98.7
	LTR (*)	124	151,145	0.151	617.5	97.75
**DNA - transposon**	Helitron (*)	267	447,385	0.446	1514.0	96.1
	Maverick (*)	14	33,884	0.034	1399.5	97.6
	MITE (**)	1,101	702,012	0.700	521.0	95.0
	TIR (**)	1,475	1,582,321	1.578	815.0	97.1
	**Total**	**3,485**	**3,613,088**	**3.603**		

Table S7: ***M. incognita*** **per-order summary of copies % identity with their consensus.**

	Min.	1st Quantile	Median	Mean	3rd Quantile	Max.
Helitron	85.3	92.0	94.4	93.4	95.8	97.7
LARD	92.6	96.1	97.1	96.9	97.9	99
LINE	85.9	95.4	96.6	96.3	98.8	100
LTR	85.3	94.8	97.0	96.3	98.4	100
Maverick	85.0	90.3	95.3	93.7	97.2	99.8
MITE	85.0	92.8	96.2	95.3	98.4	100
SINE	93.4	99.3	99.7	98.9	99.8	100
TIR	85.0	93.7	97.3	96.0	99.2	100
TRIM	85.2	95.7	97.7	96.8	98.8	99.9

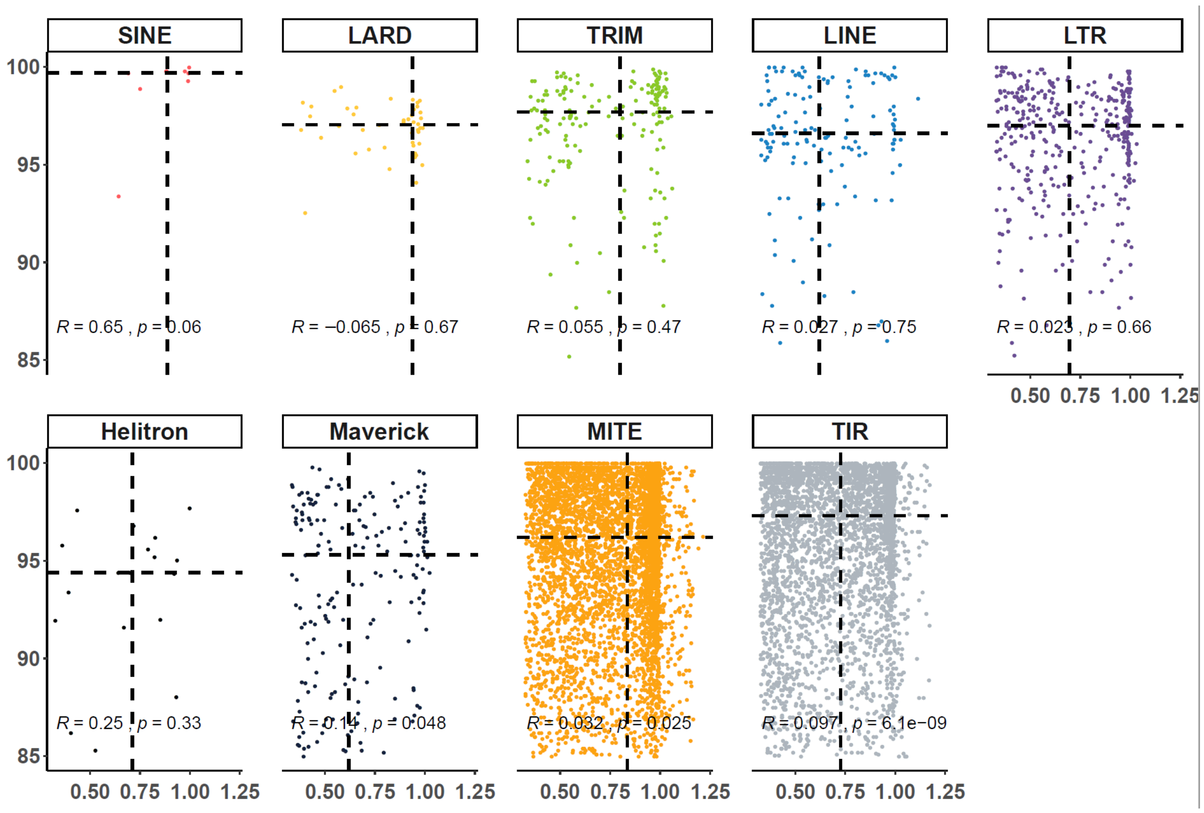

Fig S5: ***M. incognita*** **per TE copy % identity with its consensus in function of the proportion of consensus covered.**

Data are splitted in panels according to TE orders. For each panel, Y-axis represents the percentage of identity a copy shares with its consensus. X-axis represents the coverage of the TE consensus (proportion). Coverage values > 100% correspond to cases for which the copy includes a nested sequence regarding the TE consensus sequence (other TE, repeats, other). Each point represents a TE locus (*i.e.* a TE copy). Dashed lines represent the per order median value of both the identity percentage (horizontal line) and the proportion of coverage (vertical line). R value represents the correlation coefficient (Pearson) computed for each order, and p is the associated p-value.

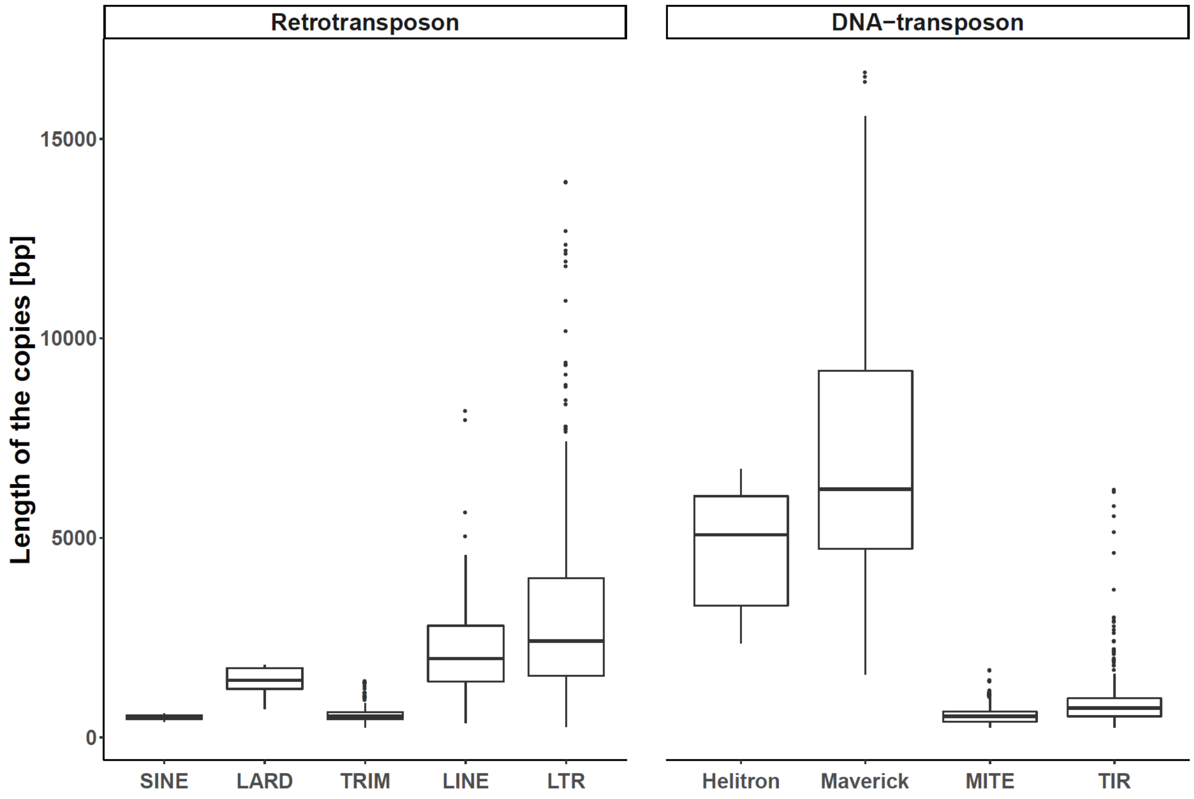

Fig S6: Distribution of TE lengths per-order (*M. incognita*).

Box plots per order of the distribution of the canonical TE annotations' length .

Table S8: canonical TE annotations with putative transposition machinery

	autonomous (*) / non-autonomous (**) orders	nb. of annotations with putative transposition machinery	nb. of annotations with substantially expressed putative transposition machinery
**retro - transposon**	SINE (**)	0	0
	LARD (**)	0	0
	TRIM (**)	0	0
	LINE (*)	54	26
	LTR (*)	147	45
**DNA - transposon**	Helitron (*)	17	3
	Maverick (*)	63	26
	MITE (**)	0	0
	TIR (*)	30	6

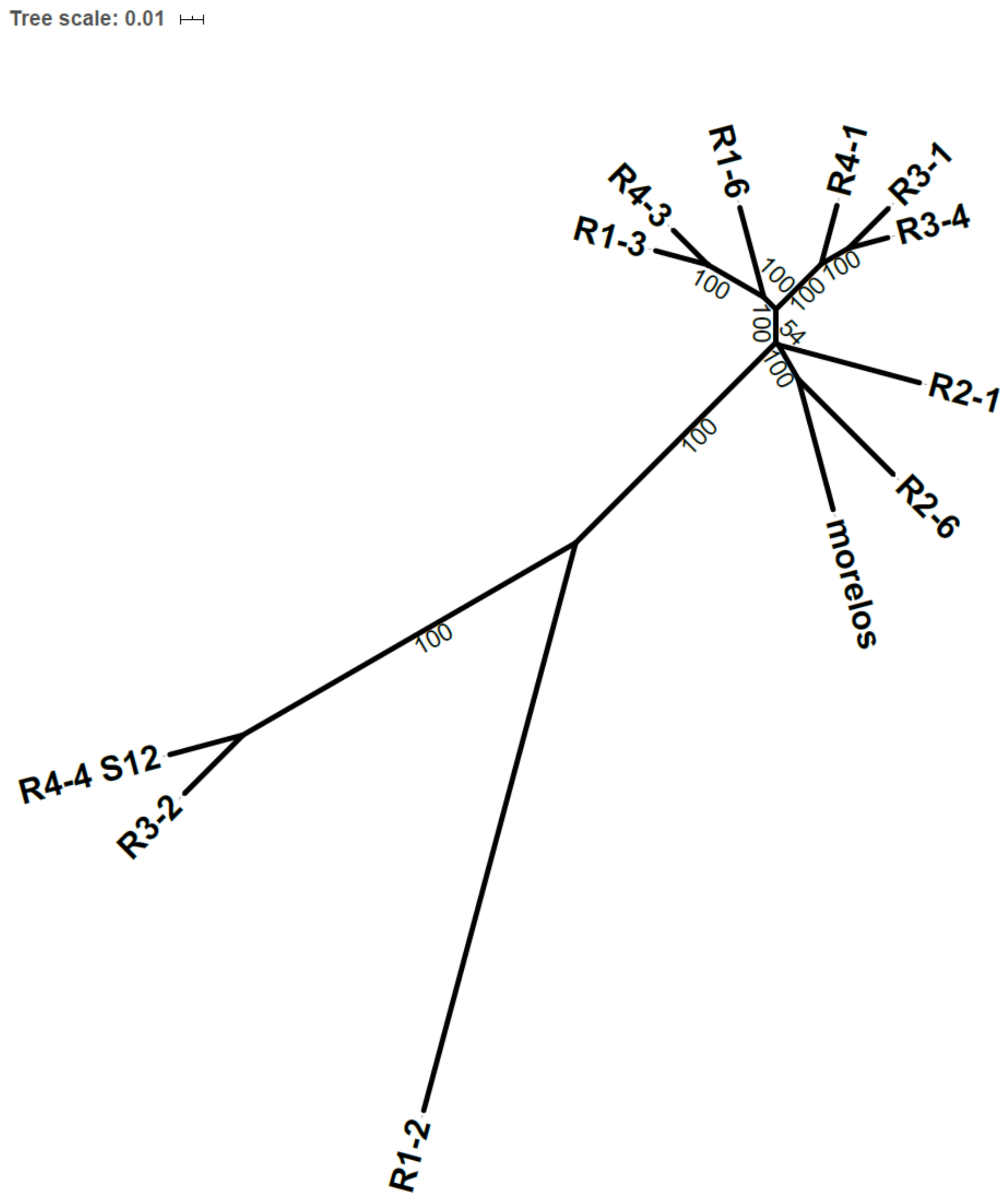

Tree scale: 0.01
R4-3
R1-6
R4-1
R3-1
R3-4
R1-3
100
100
100
100
100
54
100
R2-1
100
R2-6
morelos
100
R4-4 S12
R3-2
R1-2

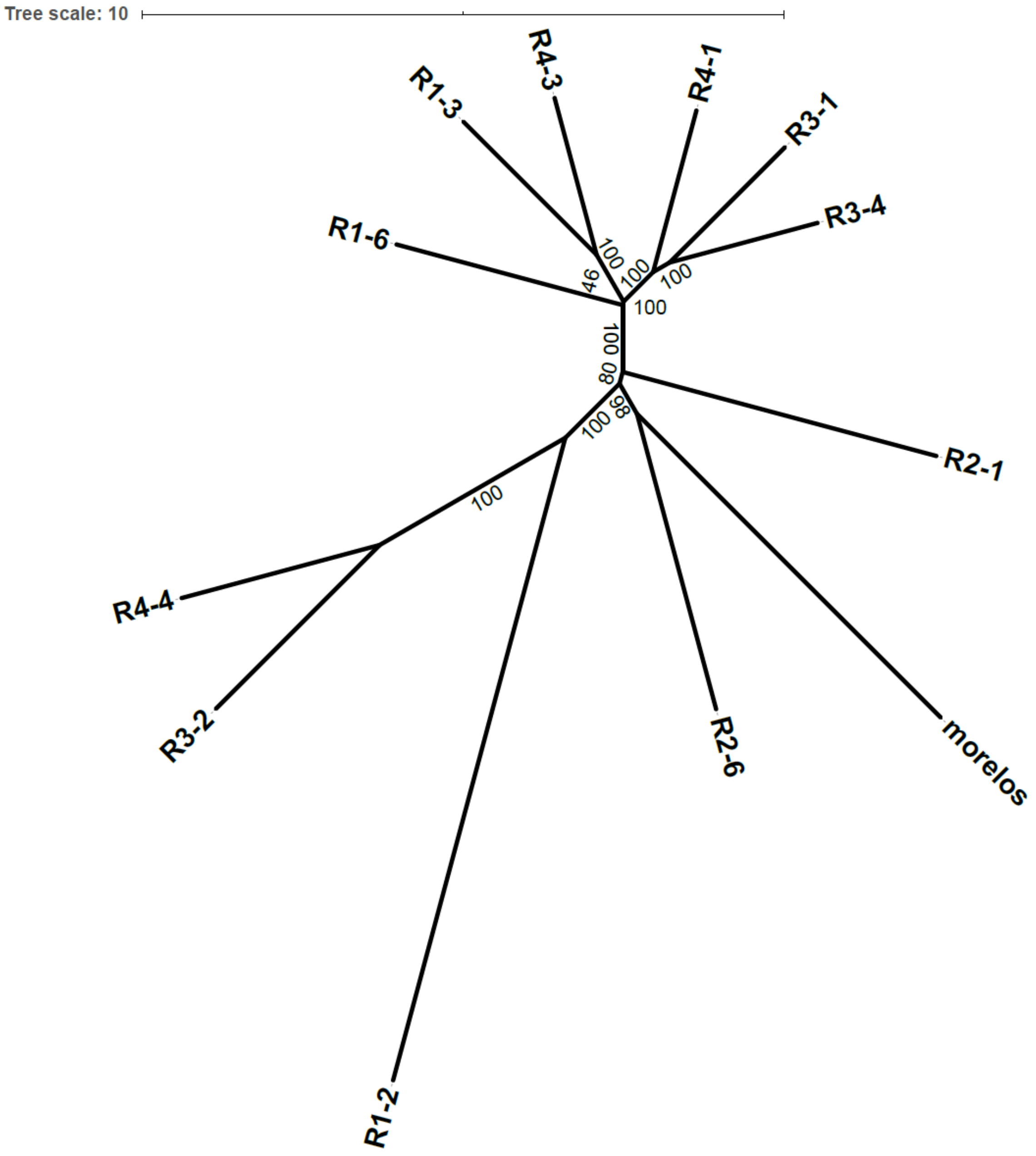

Fig S7: Phylogenetic tree for *M. incognita* isolates with branch length.

Top Phylogenetic tree is based on SNP present in coding sequences. Maximum Likelihood (ML) tree reconstruction. Bottom Phylogenetic tree based on TE-frequencies euclidean distances between isolates. Neighbor-Joining (NJ) tree reconstruction. For both trees, the branch length is displayed. The scale is displayed in the top-left corner of each sub-figure. Values in black (bootstrap values) represent branch support values, 100 being the maximal value.

Table S9: TE repartition per orders in the reference annotation and between polymorphisms types.

Ref-annotation line represents the per-order number of elements in the reference genome annotation. The sum of "non-polymorphic ref." and "polymorphic-ref" is not equal to the number of reference annotations due to filtering criteria. See sup. Fig S1 for detailed explanations.

	SINE	LARD	TRIM	LINE	LTR	Helitron	Maverick	MITE	TIR
**ref-annotations**	9	45	174	145	373	18	189	5085	3595
**non-polymorphic ref-annotations**	8	35	154	128	322	16	179	3602	2657
**polymorphic ref-annotations**	1	6	14	13	37	1	7	1194	818
**new**	0	0	0	10	11	0	0	192	74
**unannotated**	0	0	4	6	12	2	16	97	69

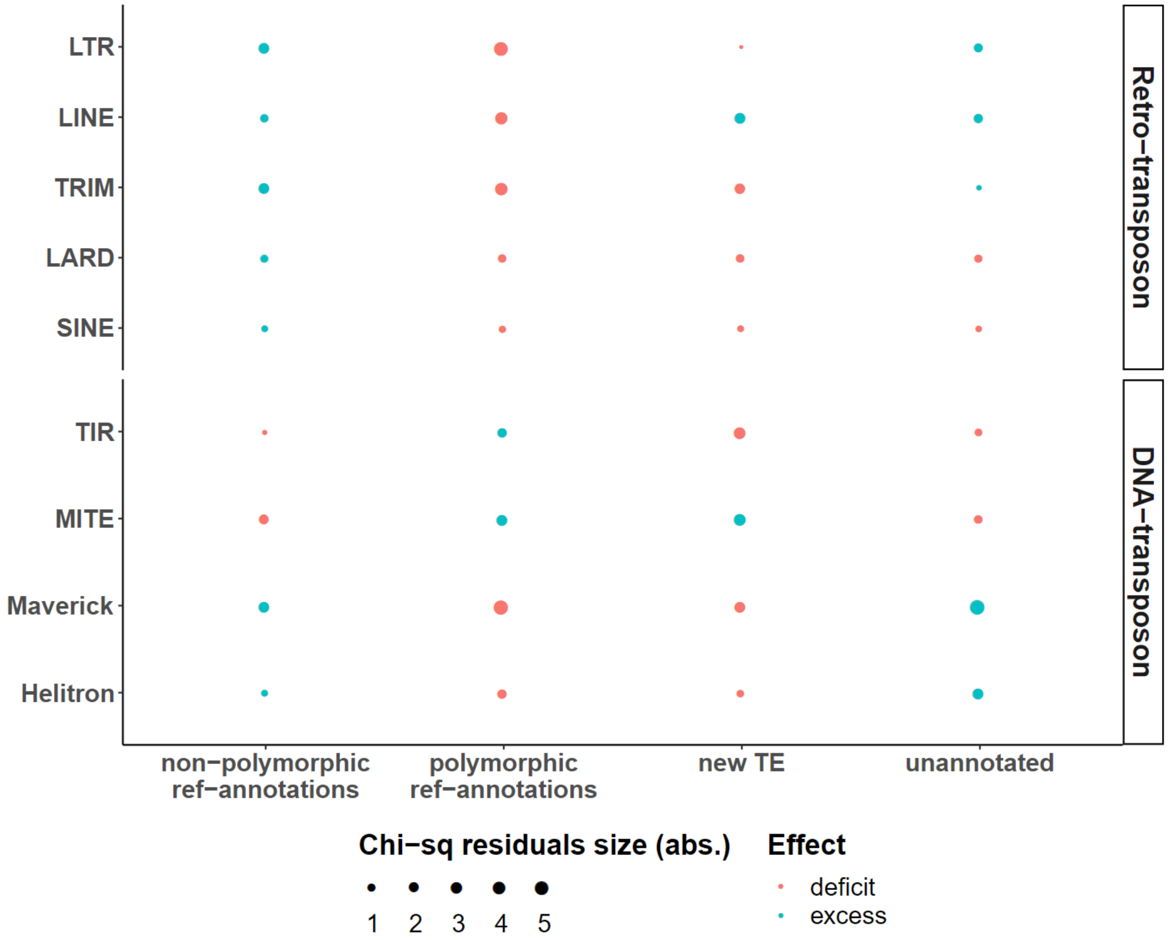

Fig S8: Relative abundance of the TE copies (count) per polymorphism types and TE-orders.

Each point represents a chi-square residual value. Chi-square residuals are the distance from the expected distribution under the homogeneity hypothesis. They are used here as a proxy to estimate the relative abundance (count) per polymorphism type and TE order and how each combination differs from the expected distribution. For each point, the wider is the surface, the higher is the distance from the expectation. Red points represent a deficit compared to the expectation while the blue points represent an excess.

Table S10: Number of TE copies per-consensus for HCP loci.

consensus	order	nb. of HCPTEs copies
DTX-comp_mincV3XDN-B-R1459-Map20	TIR	8
DTX-incomp_mincV3XDN-B-R11531-Map10	TIR	2
DTX-incomp_mincV3XDN-B-R271-Map10	TIR	1
DTX-incomp_mincV3XDN-B-R3892-Map20	TIR	1
DXX-MITE_mincV3XDN-B-G1048-Map15	MITE	1
DXX-MITE_mincV3XDN-B-G305-Map9	MITE	1
DXX-MITE_mincV3XDN-B-R14125-Map7	MITE	1
DXX-MITE_mincV3XDN-B-R306-Map20	MITE	10
DXX-MITE_mincV3XDN-B-R321-Map20	MITE	1
DXX-MITE_mincV3XDN-B-R3266-Map20	MITE	1
DXX-MITE_mincV3XDN-B-R3611-Map9	MITE	4
RIX-comp_mincV3XDN-B-R6875-Map20_reversed	LINE	1
RIX-incomp_mincV3XDN-B-R4613-Map9	LINE	1

Table S11: Orthologs to genes potentially impacted by TE neo-insertions.

Entries with bold font are *M. incognita* genes with orthologs in other *Meloidogyne species* or other Plant Parasitic Nematode (PPN) genus only. Entries are sorted by gene name. Genes highlighted in yellow are the genes potentially impacted by neo-insertions which have been selected for experimental validation.

Gene	Wormbase gene trees orthologs	Which species	Tree URL
Minc3s00005g00347	154	in many nematodes and other animals	https://parasite.wormbase.org/Meloidogyne_incognita_prjeb8714/Gene/Compara_Tree?g=Minc3s00005g00347;r=FXSY01000005.1:267231-279946;t=Minc3s00005g00347
Minc3s00005g00348	168	in many nematodes and other animals	https://parasite.wormbase.org/Meloidogyne_incognita_prjeb8714/Gene/Compara_Tree?g=Minc3s00005g00348;r=FXSY01000005.1:271509-282281;t=Minc3s00005g00348
**Minc3s00026g01668**	14	**Meloidogyne-specific: incognita, arenaria, javanica, floridensis**	https://parasite.wormbase.org/Meloidogyne_incognita_prjeb8714/Gene/Compara_Tree?g=Minc3s00026g01668;r=FXSY01000026.1:125149-126932;t=Minc3s00026g01668;collapse=
Minc3s00137g05752	122	in many nematodes and other animals	https://parasite.wormbase.org/Meloidogyne_incognita_prjeb8714/Gene/Compara_Tree?g=Minc3s00137g05752;r=FXSY01000137.1:70079-73404;t=Minc3s00137g05752
Minc3s00157g06330	135	in many nematodes and other animals	https://parasite.wormbase.org/Meloidogyne_incognita_prjeb8714/Gene/Compara_Tree?g=Minc3s00157g06330;r=FXSY01000157.1:83470-88312;t=Minc3s00157g06330
Minc3s00199g07364	203	in many nematodes and other animals	https://parasite.wormbase.org/Meloidogyne_incognita_prjeb8714/Gene/Compara_Tree?g=Minc3s00199g07364;r=FXSY01000199.1:14729-17937;t=Minc3s00199g07364
Minc3s00199g07365	149	nematode specific but many nematodes	https://parasite.wormbase.org/Meloidogyne_incognita_prjeb8714/Gene/Compara_Tree?g=Minc3s00199g07365;r=FXSY01000199.1:18300-23780;t=Minc3s00199g07365
Minc3s00201g07425	188	in many nematodes and other animals	https://parasite.wormbase.org/Meloidogyne_incognita_prjeb8714/Gene/Compara_Tree?g=Minc3s00201g07425;r=FXSY01000201.1:30179-31671;t=Minc3s00201g07425
Minc3s00201g07426		tRNA (non-coding), widely conserved in nematodes	
Minc3s00201g07427	5	nematode specific, mainly Meloidogyne but also Chromadorea and Dirofilaria	https://parasite.wormbase.org/Meloidogyne_incognita_prjeb8714/Gene/Compara_Tree?g=Minc3s00201g07427;r=FXSY01000201.1:31822-32328;t=Minc3s00201g07427

Minc3s00301g09724	129	in many nematodes and other animals	https://parasite.wormbase.org/Meloidogyne_incognita_prjeb8714/Gene/Compara_Tree?g=Minc3s00301g09724;r=FXSY01000301.1:27845-35780;t=Minc3s00301g09724
**Minc3s00450g12515**	**5**	**Meloidogyne-specific: incognita, arenaria.**	https://parasite.wormbase.org/Meloidogyne_incognita_prjeb8714/Gene/Compara_Tree?g=Minc3s00450g12515;r=FXSY01000450.1:51949-52954;t=Minc3s00450g12515;collapse=
**Minc3s00621g15225**	9	**Meloidogyne-specific: incognita, arenaria, javanica, floridensis, enterolobii, hapla, graminicola.**	https://parasite.wormbase.org/Meloidogyne_incognita_prjeb8714/Gene/Compara_Tree?g=Minc3s00621g15225;r=FXSY01000621.1:38374-38735;t=Minc3s00621g15225
Minc3s00667g15847	17	nematode specific, all Plant Parasitic Nematodes (PPN) except A. nanus	https://parasite.wormbase.org/Meloidogyne_incognita_prjeb8714/Gene/Compara_Tree?g=Minc3s00667g15847;r=FXSY01000667.1:10892-13619;t=Minc3s00667g15847
**Minc3s00751g16867**	13	**Meloidogyne-specific: incognita, arenaria, javanica, floridensis, enterolobii.**	https://parasite.wormbase.org/Meloidogyne_incognita_prjeb8714/Gene/Compara_Tree?g=Minc3s00751g16867;r=FXSY01000751.1:15531-16499;t=Minc3s00751g16867
Minc3s00905g18730	251	in many nematodes and other animals	https://parasite.wormbase.org/Meloidogyne_incognita_prjeb8714/Gene/Compara_Tree?g=Minc3s00905g18730;r=FXSY01000905.1:1630-5121;t=Minc3s00905g18730
**Minc3s00905g18731**	5	**Meloidogyne-specific: incognita, arenaria, javanica, floridensis, enterolobii.**	https://parasite.wormbase.org/Meloidogyne_incognita_prjeb8714/Gene/Compara_Tree?g=Minc3s00905g18731;r=FXSY01000905.1:5454-6420;t=Minc3s00905g18731
**Minc3s00909g18773**	14	**Meloidogyne-specific: incognita, arenaria, javanica, floridensis, enterolobii, graminicola.**	https://parasite.wormbase.org/Meloidogyne_incognita_prjeb8714/Gene/Compara_Tree?g=Minc3s00909g18773;r=FXSY01000909.1:23309-24625;t=Minc3s00909g18773
Minc3s00965g19365	160	in many nematodes and other animals	https://parasite.wormbase.org/Meloidogyne_incognita_prjeb8714/Gene/Compara_Tree?g=Minc3s00965g19365;r=FXSY01000965.1:6357-15984;t=Minc3s00965g19365
**Minc3s00988g19605**	3	**Meloidogyne-specific: incognita, arenaria, javanica.**	https://parasite.wormbase.org/Meloidogyne_incognita_prjeb8714/Gene/Compara_Tree?g=Minc3s00988g19605;r=FXSY01000988.1:15653-17968;t=Minc3s00988g19605
**Minc3s01127g20975**	4	**Meloidogyne-specific: incognita, arenaria, javanica, floridensis, hapla.**	https://parasite.wormbase.org/Meloidogyne_incognita_prjeb8714/Gene/Compara_Tree?g=Minc3s01127g20975;r=FXSY01001127.1:7481-15500;t=Minc3s01127g20975
**Minc3s01138g21099**	6	**Meloidogyne-specific: incognita, arenaria, javanica, floridensis, enterolobii, hapla, graminicola.**	https://parasite.wormbase.org/Meloidogyne_incognita_prjeb8714/Gene/Compara_Tree?g=Minc3s01138g21099;r=FXSY01001138.1:37292-39709;t=Minc3s01138g21099

Minc3s01318g22714	10	**PPN-specific: i) Meloidogyne: incognita, arenaria, javanica, floridensis, enterolobii, graminicola; ii) Globobodera: rostochiensis; iii) Ditylenchus: destructor**	https://parasite.wormbase.org/Meloidogyne_incognita_prjeb8714/Gene/Compara_Tree?g=Minc3s01318g22714;r=FXSY01001318.1:1931-3523;t=Minc3s01318g22714
Minc3s01455g23950	0	no gene tree at all: gene specific to Meloidogyne incognita	
**Minc3s01827g26567**	3	**Meloidogyne-specific: incognita, arenaria, javanica, floridensis, enterolobii.**	https://parasite.wormbase.org/Meloidogyne_incognita_prjeb8714/Gene/Compara_Tree?g=Minc3s01827g26567;r=FXSY01001827.1:2587-2859;t=Minc3s01827g26567;collapse=9293401
Minc3s02496g30324	170	in many nematodes and other animals	https://parasite.wormbase.org/Meloidogyne_incognita_prjeb8714/Gene/Compara_Tree?g=Minc3s02496g30324;r=FXSY01002496.1:10099-17217;t=Minc3s02496g30324
Minc3s03567g34213	78	Present in many animals then only Meloidogyne	https://parasite.wormbase.org/Meloidogyne_incognita_prjeb8714/Gene/Compara_Tree?g=Minc3s03567g34213;r=FXSY01003567.1:9085-10645;t=Minc3s03567g34213;collapse=14989368.14989316.14989313.14989310.14988329

Table S12: pairwise blastn of locus 1 sequencing results.

insertion predicted	subject	query	sequence	%identity	query cover (%)	e-value	average % identity
N	morelos	R2-1	F	98.68	99	6,00E-75	99.34
			R	100	91	2,00E-73	
N	morelos	R2-6	F	100	93	8,00E-73	95.3
			R	90.6	89	4,00E-52	
N	R2-6	R2-1	F	98.64	96	1,00E-71	94.45
			R	90.26	92	4,00E-52	
Y	R1-2	R4-4	F	92.73	72	0,00E+00	96.25
			R	99.77	99	0,00E+00	
Y	R3-2	R1-2	F	98.52	98	0,00E+00	98.88
			R	99.24	89	0,00E+00	
Y	R4-4	R3-2	F	92.16	93	0,00E+00	95.7
			R	99.24	98	0,00E+00	

References

Koutsovoulos GD, Marques E, Arguel M-J, Duret L, Machado ACZ, Carneiro RMDG, Kozlowski DK, Bailly-Bechet M, Castagnone-Sereno P, Albuquerque EVS, et al. 2020. Population genomics supports clonal reproduction and multiple independent gains and losses of parasitic abilities in the most devastating nematode pest. *Evol. Appl.* 13:442–457.

Kozlowski D. 2020. TE polymorphisms detection and analysis with PopoolationTE2. *Portail Data INRAE* [Internet]. Available from: https://doi.org/10.15454/EWJCT8
